# Sensor-Based Assistive Devices for Visually-Impaired People: Current Status, Challenges, and Future Directions

**DOI:** 10.3390/s17030565

**Published:** 2017-03-10

**Authors:** Wafa Elmannai, Khaled Elleithy

**Affiliations:** Department of Computer Science and Engineering, University of Bridgeport, Bridgeport, CT 06604, USA; welmanna@my.bridgeport.edu

**Keywords:** assistive devices, visually-impaired people, obstacles detection, navigation and orientation systems, obstacles avoidance

## Abstract

The World Health Organization (WHO) reported that there are 285 million visually-impaired people worldwide. Among these individuals, there are 39 million who are totally blind. There have been several systems designed to support visually-impaired people and to improve the quality of their lives. Unfortunately, most of these systems are limited in their capabilities. In this paper, we present a comparative survey of the wearable and portable assistive devices for visually-impaired people in order to show the progress in assistive technology for this group of people. Thus, the contribution of this literature survey is to discuss in detail the most significant devices that are presented in the literature to assist this population and highlight the improvements, advantages, disadvantages, and accuracy. Our aim is to address and present most of the issues of these systems to pave the way for other researchers to design devices that ensure safety and independent mobility to visually-impaired people.

## 1. Introduction

The World Health Organization (WHO) Fact reported that there are 285 million visually-impaired people worldwide. Among these individuals, there are 39 million who are blind in the world [1]. More than 1.3 million are completely blind and approximately 8.7 million are visually-impaired in the USA [2]. Of these, 100,000 are students, according to the American Foundation for the Blind [2] and National Federation for the Blind [3]. Over the past years, blindness that is caused by diseases has decreased due to the success of public health actions. However, the number of blind people that are over 60 years old is increasing by 2 million per decade. Unfortunately, all these numbers are estimated to be doubled by 2020 [4].

The need for assistive devices for navigation and orientation has increased. The simplest and the most affordable navigations and available tools are trained dogs and the white cane [5]. Although these tools are very popular, they cannot provide the blind with all information and features for safe mobility, which are available to people with sight [6,7].

### 1.1. Assistive Technology

All the systems, services, devices and appliances that are used by disabled people to help in their daily lives, make their activities easier, and provide a safe mobility are included under one umbrella term: assistive technology [8].

In the 1960s, assistive technology was introduced to solve the daily problems which are related to information transmission (such as personal care) [9], navigation and orientation aids which are related to mobility assistance [10,11,12].

**Figure 1:**
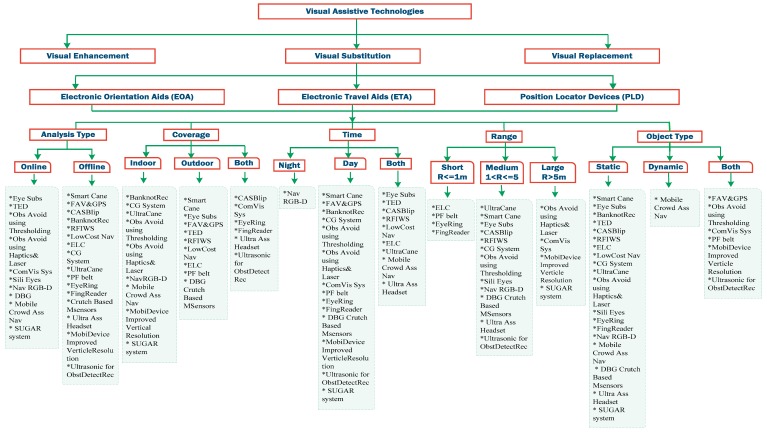
Classification of electronic devices for visually-impaired people.

In Figure 1, visual assistive technology is divided into three categories: vision enhancement, vision substitution, and vision replacement [12,13]. This assistive technology became available for the blind people through electronic devices which provide the users with detection and localization of the objects in order to offer those people with sense of the external environment using functions of sensors. The sensors also aid the user with the mobility task based on the determination of dimensions, range and height of the objects [6,14].

The vision replacement category is more complex than the other two categories; it deals with medical and technology issues. Vision replacement includes displaying information directly to the visual cortex of the brain or through an ocular nerve [12]. However, vision enhancement and vision substitution are similar in concept; the difference is that in vision enhancement, the camera input is processed and then the results will be visually displayed. Vision substitution is similar to vision enhancement, yet the result constitutes non-visual display, which can be vibration, auditory or both based on the hearing and touch senses that can be easily controlled and felt by the blind user.

The main focus in this literature survey is the vision substitution category including its three subcategories; Electronic Travel Aid (ETAs), Electronic Orientation Aid (EOAs), and Position Locator Devices (PLDs). Our in-depth study of all the devices that provide the after mentioned services allows us to come up with a fair taxonomy that can classify any proposed technique among others. The Classification of electronic devices for visually-impaired people is shown in Figure 1. Each one of the three categories tries to enhance the blind people’s mobility with slight differences.

#### 1.1.1. Electronic Travel Aids (ETAs)

These are devices that gather information about the surrounding environment and transfer it to the user through sensor cameras, sonar, or laser scanners [15,16]. The rules of ETAs according to the National Research Council [6] are:

(1) Determining obstacles around the user body from the ground to the head;
(2) Affording some instructions to the user about the movement surface consists of gaps or textures;
(3) Finding items surrounding the obstacles;
(4) Providing information about the distance between the user and the obstacle with essential direction instructions;
(5) Proposing notable sight locations in addition to identification instructions;
(6) Affording information to give the ability of self-orientation and mental map of the surroundings.

#### 1.1.2. Electronic Orientation Aids (EOAs)

These are devices that provide pedestrians with directions in unfamiliar places [17,18]. The guidelines of EOAs are given in [18]:

(1) Defining the route to select the best path;
(2) Tracing the path to approximately calculate the location of the user;
(3) Providing mobility instructions and path signs to guide the user and develop her/his brain about the environment.

#### 1.1.3. Position Locator Devices (PLD)

These are devices that determine the precise position of its holder such as devices that use GPS technology.

Our focus in this paper is on the most significant and latest systems that provide critical services for visually-impaired people including obstacle detection, obstacle avoidance and orientation services containing GPS features.

In Section 2, a brief description is provided for the most significant electronic devices. Analysis of the main features for each studied device is presented in Section 3. Section 4 concludes this review with discussion about the systems’ evaluation. The final section includes future directions.

## 2. The Most Significant Electronic Devices for Visually-impaired People

Most electronic aids that provide services for visually-impaired people depend on the data collected from the surrounding environment (via either laser scanner, cameras sensors, or sonar) and transmitted to the user either via tactile, audio format or both. Different opinions on which one is a better feedback type are discussed, and this is still an open topic.

However, regardless of the services that are provided by any particular system, there are some basic features required in that system to offer a fair performance. These features can be the key to measuring the efficiency and reliability of any electronic device that provides navigation and orientation services for visually-impaired people. Therefore, we present in this section a list of the most important and latest systems with a brief summary including: what is the system, its prototype, briefly how it works, the well-known techniques that being used in that system, and the advantages and disadvantages. Those devices are classified in Figure 1 based on the described features in Table 1. The comparative results based on these features will be represented in the following section with an answer to the question of which device is the most efficient and desirable.

● *Smart Cane*

**Figure 2:**
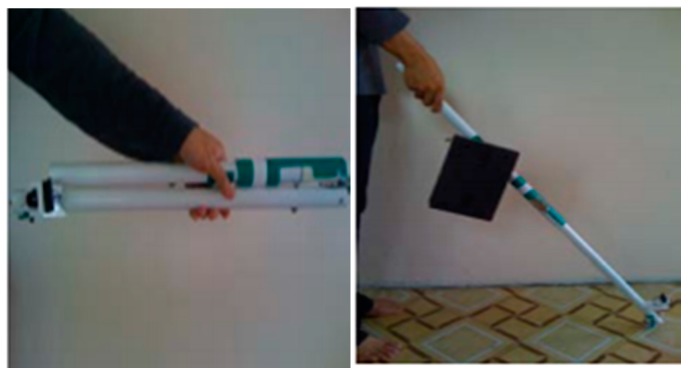
The Smart Cane prototype [19].

Wahab et al. studied the development of the Smart Cane product for detecting the objects and produce accurate instructions for navigation [19]. The Smart Cane was presented originally by Central Michigan University’s students. The design of the Smart Cane is shown in Figure 2. It is a portable device that is equipped with a sensor system. The system consists of ultrasonic sensors, microcontroller, vibrator, buzzer, and water detector in order to guide visually-impaired people. It uses servo motors, ultrasonic sensors, and fuzzy controller to detect the obstacles in front of the user and then provide instructions through voice messages or hand vibration.

The servo motors are used to give a precise position feedback. Ultrasonic sensors are used for detecting the obstacles. Hence, the fuzzy controller is able to give the accurate decisions based on the information received from the servo motors and ultrasonic sensors to navigate the user.

The output of the Smart Cane depends on gathering the above information to produce audio messages through the speaker to the user. In addition, hearing impaired people have special vibrator gloves that are provided with the Smart Cane. There is a specific vibration for each finger, and each one has a specific meaning.

The Smart Cane has achieved its goals in detecting the objects and obstacles, producing the needed feedback. As shown in Figure 2, the Smart Cane is easily carried and easily bent. In addition, the water sensor will not detect the water unless it is 0.5 cm or deeper and the buzzer of water detector will not stop before it is dried or wiped. The authors of the paper have some recommendations for the tested system. They stated that in order to monitor the power status, it would better to have a power supply meter being installed. The authors recommended adding a buzzer timer to specify the period to solve the buzzer’s issue as well.

● *Eye Substitution*

**Figure 3:**
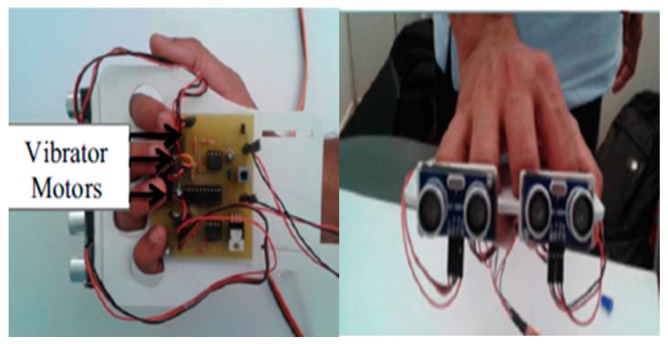
The prototype of the eye substitution device [20].

Bharambe et al. developed an embedded device to act as an eye substitution for the vision impaired people (VIP) that helps in directions and navigation as shown in Figure 3 [20]. Mainly, the embedded device is a TI MSP 430G2553 micro-controller (Texas Instruments Incorporated, Dallas, TX, USA). The authors implemented the proposed algorithms using an Android application. The role of this application is to use GPS, improved GSM, and GPRS to get the location of the person and generate better directions. The embedded device consists of two HC-SR04 ultrasonic sensors (Yuyao Zhaohua Electric Appliance Factory, Yuyao, China), and three vibrator motors.

**Figure 4:**
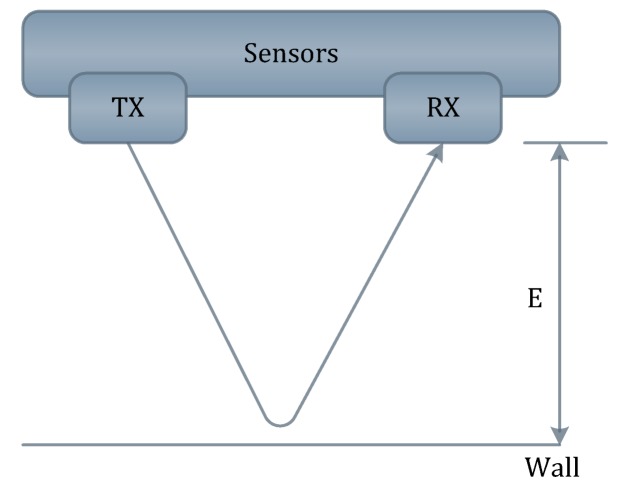
Reflection of sequence of ultrasonic pulses between the sender and receiver.

The ultrasonic sensors send a sequence of ultrasonic pulses. If the obstacle is detected, then the sound will be reflected back to the receiver as shown in Figure 4. The micro-controller processes the readings of the ultrasonic sensors in order to activate the motors by sending pulse width modulation. It also provides a low power consumption [21].

**Figure 5:**
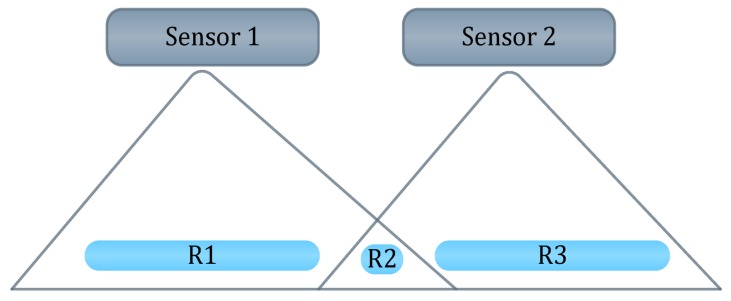
Ranges that are covered by ultra-sonic sensors [20].

The design of the device is light and very convenient. Furthermore, the system uses two sensors to overcome the issue of narrow cone angle as shown in Figure 5. So, instead of covering two ranges, the ultrasonic devices cover three ranges. This does not only help in detecting obstacles, but also in locating them. However, the design could be better if the authors did not use the wood foundation that will be carried by the user most of the time. In addition, the system is not reliable and is limited to Android devices.

● *Fusion of Artificial Vision and GPS (FAV&GPS)*

**Figure 6:**
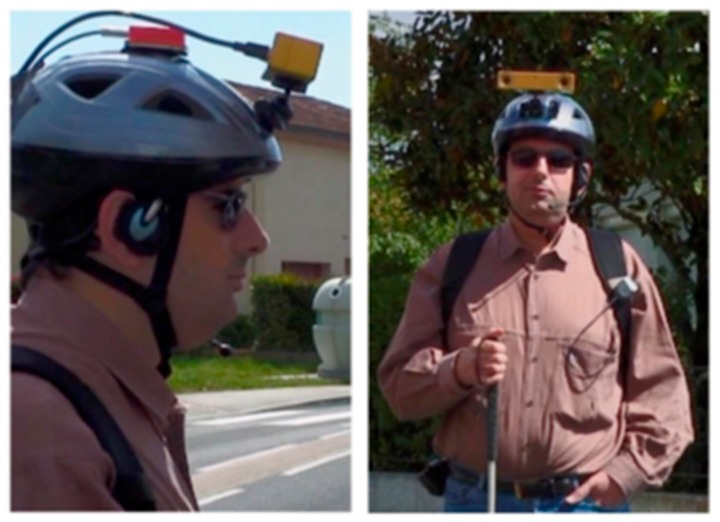
An assistive device for blind people based on a map matching approach and artificial vision [22].

An assistive device for blind people was introduced in [22] to improve mapping of the user’s location and positioning the surrounding objects using two functions that are: based on a map matching approach and artificial vision [23]. The first function helps in locating the required object as well as allowing the user to give instructions by moving her/his head toward the target. The second one helps in automatic detection of visual aims. As shown in Figure 6, this device is a wearable device that mounted on the user’s head, and it consists of two Bumblebee stereo cameras for video input that installed on the helmet, GPS receiver, headphones, microphone, and Xsens Mti tracking device for motion sensing. The system processes the video stream using SpikNet recognition algorithm [24] to locate the visual features that handle the 320 × 240 pixels image.

For fast localization and detection of such visual targets, this system integrated Global Position System (GPS), modified Geographical Information System (GIS) [25] and vision based positioning. This design is able to improve the performance of the navigation systems where the signal is deputized. Therefore, this system can be combined with any navigation system to overcome the issues of the navigation in such areas.

**Figure 7:**
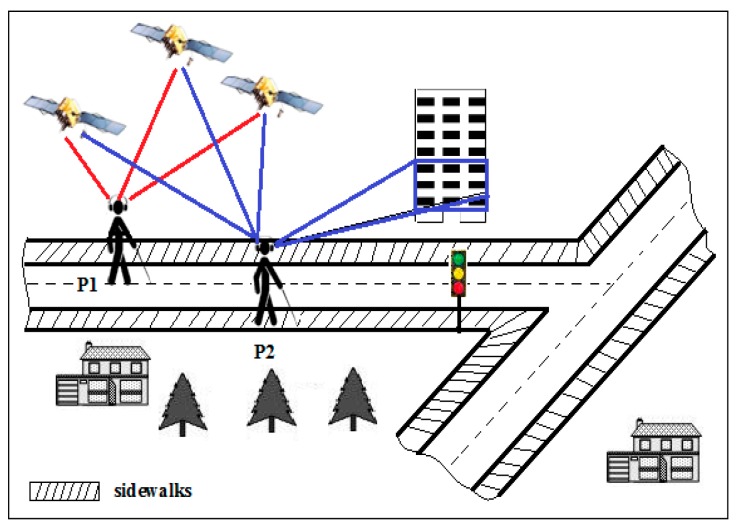
The result of mapping both commercial Geographical Information System (GIS) and Global Position System (GPS)’s signals is P1. P2 is the result of mapping the signals of GPS with adapting GIS [22].

Due to the lack of the availability of some information about the consistency of pedestrian mobility by commercial GIS, this system maps the GPS signal with the adapting GIS to estimate the user’s current position as shown in Figure 7. The 3D target’s position is calculated using matrices of lenses and stereoscopic variance. After detecting the user and target positions, the vision agent sends the ID of the target and its 3D coordinates.

The matrix of the rotation of each angle is multiplied with the target coordinates in the head reference frame (*x*, *y*, *z*) to obtain the targets’ coordinates in the map reference (*x*^−^, *y*^−^, *z*^−^) as given in Equation (1). After that, the design uses Geographic Information System (GIS) that contain all targets goelocated positions to get the longitude and latitude of landmarks. Based on this information, the authors could compute the user’s coordinates in World Geodetic System Coordinates (WGS84). The results are in audio format through the speaker that is equipped with the device.

(1) $$\begin{array}{c} \left[\begin{array}{c} x^{\prime }\\ y^{\prime }\\ z^{\prime } \end{array}\right]=\left[\begin{array}{c} x\\ y\\ z \end{array}\right]\cdot \left[\begin{array}{ccc} 1 & 0 & 0\\ 0 & \cos\left(yaw\right) & \sin\left(yaw\right)\\ 0 & \sin\left(yaw\right) & \cos\left(yaw\right) \end{array}\right].\\ \left[\begin{array}{ccc} \cos\left(pitch\right) & 0 & -\sin\left(pitch\right)\\ 0 & 1 & 0\\ \sin\left(pitch\right) & 0 & \cos\left(pitch\right) \end{array}\right].\left[\begin{array}{ccc} \cos\left(roll\right) & \sin\left(roll\right) & 0\\ \sin\left(roll\right) & \cos\left(roll\right) & 0\\ 0 & 0 & 1 \end{array}\right] \end{array}$$

The use of the modified GIS shows positive results and better estimation of the user’s position compared to the commercial GIS as shown in Figure 7. However, the system has not been tested on navigation systems to insure its performance if it is integrated with a navigation system. So, whether it will enhance the navigation systems or not is unknown.

● *Banknote Recognition (BanknoteRec)*

An assistive device for blind people was implemented in [26] to help them classify the type of banknotes and coins. The system was built based on three models: input (OV6620 Omni vision CMOS camera), process (SX28 microcontroller), and output (speaker).

RGB color model is used to specify the type of the banknote by calculating the average red, green, and blue color. The function of the microcontroller (IV-CAM) with the camera mounted on a chip is used to extract the desired data from the camera’s streaming video. Then, the mean color and the variance data will be gathered for next step when MCS-51 microcontroller starts to process this gathered information. Based on the processing results, IC voice recorder (Aplus ap8917) records the voice of each kind of banknote and coin.

This system compares some samplings of each kind of a banknote using RGB model. The best matching banknote will be the result of the system. However, the coin is identified based on the size by computing the number of pixels. To find the type of the coin, the average of pixel number of each coin needs to be calculated. The best matching resultant coin will be the result of the device through the speaker.

The accuracy of the results is 80% due to two factors; the difference of the color of the new and old currency and a different light from the nature light might affect the results. On the other hand, the device was only tested on Thai currency. Therefore, the system is not reliable, and we cannot guarantee the efficiency of the system’s performance on other types of currency. Also, the device may not identify other banknotes than the tested if each kind of the banknote have a unique color and the coins that do not have similar size.

Recently, similar work was presented in [27]. This device is a portable one that shows a reasonable accuracy in detecting the Euro banknotes with a good accuracy in recognizing it by integrating well known computer vision techniques. However, the system has a very limited scope for a particular application such as the coins were not considered for detection and recognition. Furthermore, fake banknotes are not detected by the system.

● *TED*

**Figure 8:**
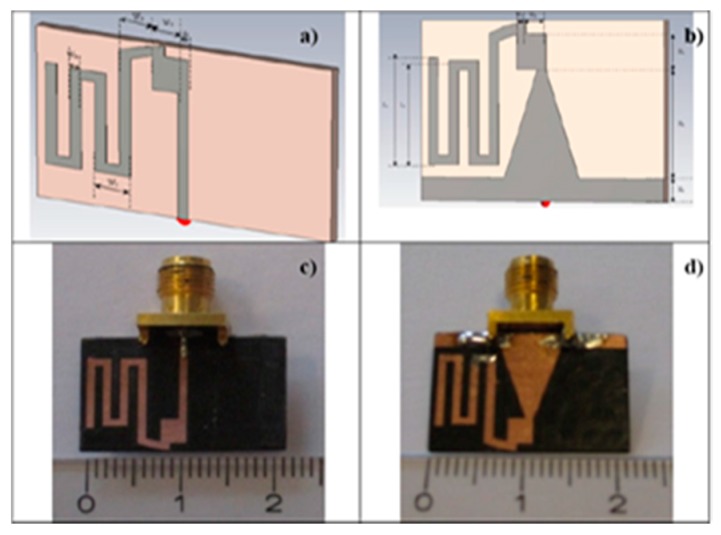
(**a**) The design of the antenna at the front and (**b**) at the back; (**c**) fabricated antenna at the front; (**d**) at the back and [30].

A design of a tiny dipole antenna was developed in [28] to be connected within Tongue-placed electro-tactile device (TED) to assist blind people in transmitting information and navigating. This antenna is designed to establish a wireless communication between the TED device and matrix of electrodes. The design of the antenna in front and the back is shown in Figure 8a–d. Bazooka Balun is used to reduce the effect of the cable on a small antenna [29].

The idea of a TED system that was later designed in [30] is a development of Paul Bach-Y-Rita system into a tiny wireless system. The visual information of all video inputs are displayed into a tactile display unit.

**Figure 9:**
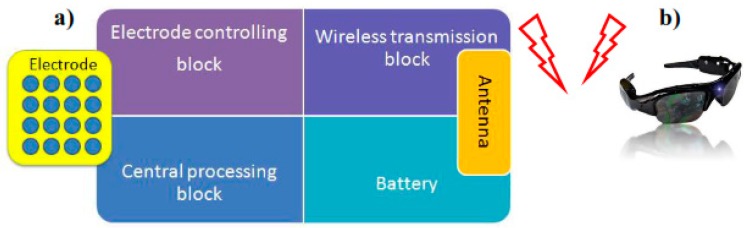
Tongue-placed electro-tactile system with sunglasses carries object detection camera [28] (**a**) sunglasses with detective camera of objects; (**b**) tongue electro tactile device.

**Figure 10:**
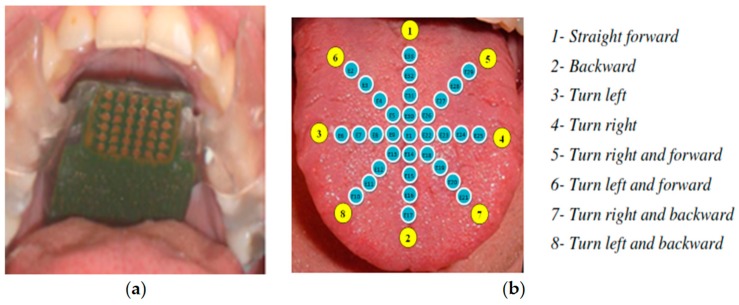
(**a**) Matrix of electrode; (**b**) Different eight directions for the matrix of electrodes [30].

The design of this system as shown in Figure 9 is based on three main parts; sunglasses with detective camera of objects, tongue electro tactile device (TED), and a host computer. The device contains an antenna to support wireless communication in the system, a matrix of electrodes to help the blind sensing through the tongue, a central processing block (CPU), a wireless transmission block, an electrode controlling block, and a battery. A matrix of 33 electrodes that is distributed into 8 pulses will be replaced into the blind person’s tongue as shown in Figure 10, and the remaining components will be fabricated into a circuit. Each pulse corresponds to a specific direction.

**Figure 11:**
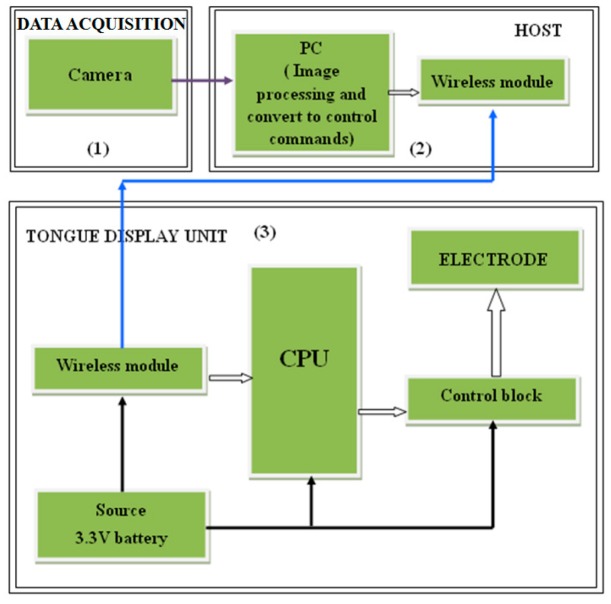
The overall design of the system [30].

The image signals that are sent from the camera to the electrodes matrix will be received by the host computer first, and then it will be transferred in interpretable information. Hence, this converted information will be received by the wireless transmission block of the TED device as shown in Figure 11. Next, the image signal will be processed into an encoded signal by the central processing block; that will be processed into controlled signal by the electrode controlling block afterwards. In the end, the controlled signal will be sent to the electrodes.

Although this device meets its goal and show an effective performance, the results show that the antenna is not completely omni-directional. It indicates that the system is not optimized and requires further tests. In addition, the device was tested on a number of blind people. The results show that the user is not responding to some of the pulses, for example, the pulse number 7. This is indicating that the system is not sending the pulse to that particular point.

● *CASBlip*

**Figure 12:**
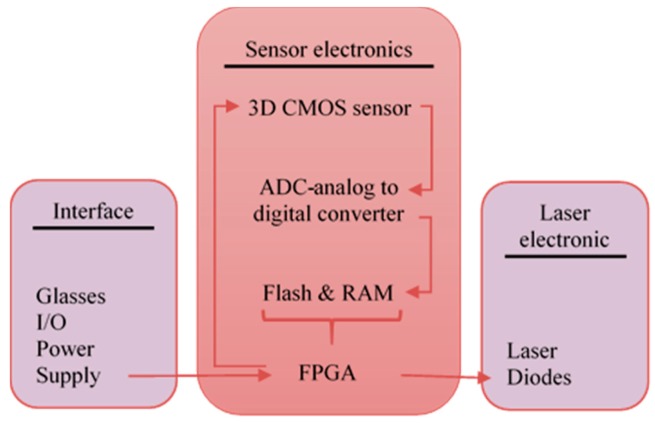
Design of the sensor module [31].

A wearable aid system for blind people (CASBlip) was proposed in [31]. The aims of this design are to provide object detection, orientation, and navigation aid for both partially and completely blind people. This system has two important modules: sensor module and acoustic module. The sensor module contains a pair of glasses that includes the 1X64 3D CMOS image sensors and laser light beams for object detection as shown in Figure 12. In addition, it has a function implemented using Field Programmable Gate Array (FPGA) that is controlling the reflection of the laser light beams after its collision with enclosure object to the lenses of the camera, calculating the distance, acquisition the data, and controlling the application software. The other function of FPGA was implemented within the acoustic module in order to process the environmental information for locating the object and convert this information to sounds that will be received by stereophonic headphones.

The developed acoustic system in [31] allows the user to choose the route and path after detecting the presence of the object and user. However, the small range of this device can cause a serious incident. The system was tested on two different groups of blind people. However, the results of outdoor experiments were not as good as the indoor experiments. This was because of the external noise. One of the recommendations to further develop this system is to use stereovision or add more sensors for improving the image acquisition.

● *RFIWS*

A Radio Frequency Identification Walking Stick (RFIWS) was designed in [32] in order to help blind people navigating on their sidewalk. This system helps in detecting and calculating the approximate distance between the sidewalk border and the blind person. A Radio Frequency Identification (RFID) is used to transfer and receive information through radio wave medium [33]. RFID tag, reader, and middle are the main components of RFID technology.

**Figure 13:**
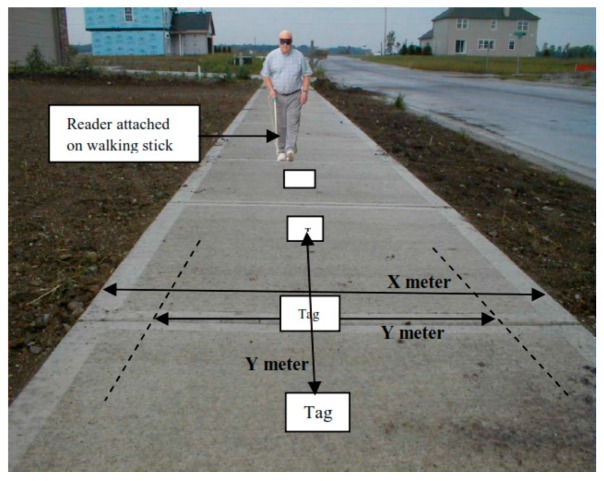
Distance of the frequency detection on sidewalk [32].

A number of RFID tags are placed in the middle of the sidewalk with consideration of an equal and specific distance between each other and RFID reader. The RFID will be connected to the stick in order to detect and process received signals. Sounds and vibrations will be produced to notify the user with the distance between the border of the sidewalk and himself/herself. Louder sounds will be generated as the user gets closer to the border. Figure 13 shows the distance of frequency detection (Y) and width of sidewalk (X). Each tag needs to be tested separately due to different ranges of detection.

RFID technology has a perfect reading function between the tags and readers that makes the device reliable in the level of detection. However, each tag needs a specific range which requires a lot of individual testing, that leads to scope limitation. Also, the system can be easily stopped from working in case of wrapping or covering the tags which prevents those tags from receiving the radio waves.

● *A Low Cost Outdoor Assistive Navigation System (LowCost Nav)*

**Figure 14:**
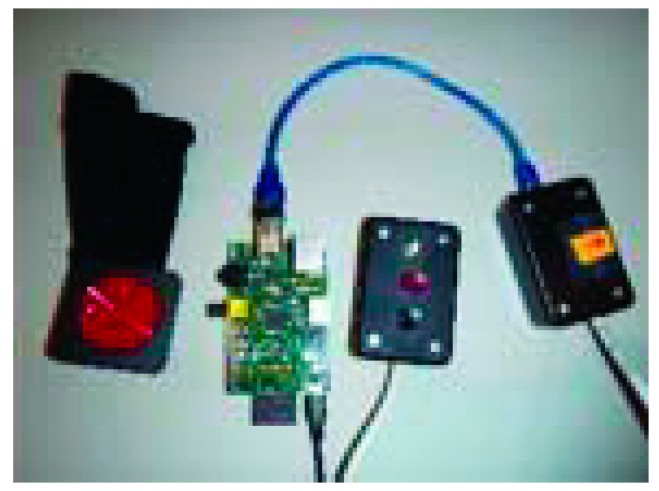
The prototype of the proposed device [34].

A navigator with 3D sound system was developed in [34] to help blind people in navigating. The device is packet on the user’s waist with Raspberry Pi, GPS receiver and three main buttons to run the system as shown in Figure 14.

The user can select a comfortable sound from recorded list to receive the navigation steps as an audible format. So, the device is provided with voice prompts and speech recognition for better capabilities. The system calculates the distance between the user and the object by using gyroscope and magnetic compass. Furthermore, the Raspberry Pi controls the process of the navigation. Both Mo Nav and Geo-Coder-US modules were used for pedestrian route generation. So, the system works as following: the user can just use the microphone to state the desired address or use one of the three provided buttons if the address already is stored in the system. User can press up button for choosing stored address, e.g., home, or entering a new address by pressing the down button and start recording the new address. The middle button will be selected to continue after the device ensure that the selected address is the correct address.

The system is composed of five main modules: loader is the controller of the system, initializer that verifies the existence of the required data and libraries, user interface that receives the desired address from the user, the address query that translates entered address to geographic coordinates, the route query obtains the user’s current location from GPS, and the route transversal that gives the audible instructions to the user to get to his destination.

**Figure 15:**
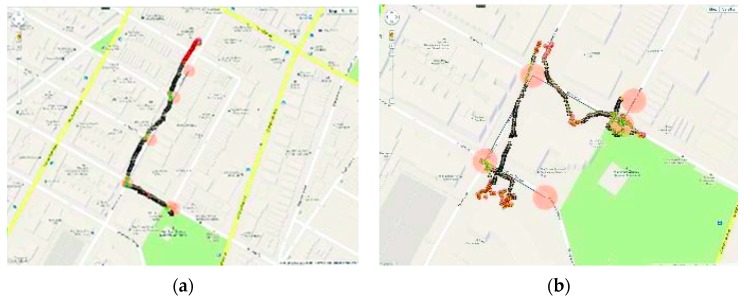
(**a**) The results of the device’s orientation in residential area; (**b**) The results of the device’s orientation in civilian [34].

This device shows a good performance within residential area as shown in Figure 15a. It is also an economically cheap for a low income people. In addition, the device is light and easy to carry. However, the device shows a low performance in civilian area where tall buildings are existence due to the low accurate performance of the used GPS receiver as shown in Figure 15b.

● *ELC*

**Figure 16:**
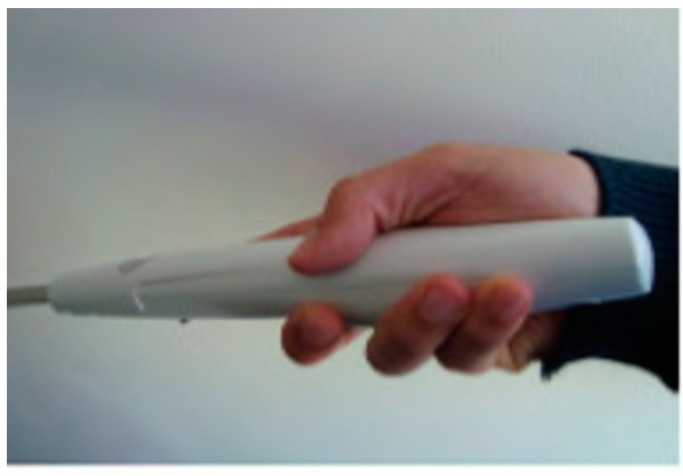
The prototype of grip [35].

**Figure 17:**
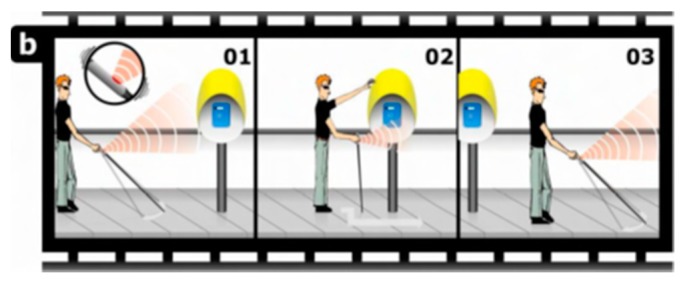
The proposed device for enhanced spatial sensitivity [35].

The proposed electronic long cane (ELC) is based on haptics technology which was presented by A.R. Garcia et al. for the mobility aid to the blind people [35]. ELC is a development of the traditional cane in order to provide an accurate detection of the objects that are around the user. A small grip of the cane shown in Figure 16 consists of an embedded electronic circuit that includes an ultrasonic sensor for detection process, micro-motor actuator as the feedback interface, and a 9 V battery as a power supplier. This grip is able to detect the obstacles above the waistline of the blind person. A tactile feedback through a vibration will be produced as warning to a close obstacle. The frequency of the feedback will be increased as the blind person gets closer to the obstacle. Figure 17 shows how the ELC could help the blind people in detecting the obstacle above his waistline, which is considered as one of the reasons to a serious injury for those who are visually-impaired or completely blind.

The ELC were tested on eight of voluntarily blind people. Physical obstacle, information obstacles, cultural obstacles are the main tested categories for the obstacles classification. The results were classified based on a taken quiz by the blind people who used the device. The results showed the efficiency of the device for physical obstacles detection above the waistline of the blind person. However, the device helps a blind person just in detecting obstacles but not in the orientation function. So, the blind person still needs to identify his path himself and relies on the tradition cane for the navigation as shown in Figure 17.

● *Cognitive Guidance System (CG System)*

Landa et al. proposed a guidance system for blind people through structured environments [36]. This design uses Kinect sensor and stereoscopic vision to calculate the distance between the user and the obstacle with help of fuzzy decision rules type Mandani and vanishing point to guide the user through the path.

The proposed system consists of two video cameras (Sony 1/3” progressive scan CCD) and one laptop. The analysis of detection range is beyond 4 m; which was obtained using stereoscopy and Kinect to compress the cloud of 3D points in range within 40 cm to 4 m in order to calculate the vanishing point. The vanishing point is used in this system as a virtual compass to direct the blind person through structured environment. Then, fuzzy decision rules are applied to avoid the obstacles.

In a first step, the system scans for planes in range between 1.5 m and 4 m. For better performance, the system processes 25 frames per second. Then the Canny filter is used for edges detection. After the edges are defined, the result is used for calculating the vanishing point. Next, the device gets the 3D Euclidean orientation from the Kinect sensor which is projected to 2D image. That gives the direction to the goal point.

This work implemented 49 fuzzy rules which cover only 80 configurations. Moreover, the vanishing point can be computed only based on existing lines which rarely exist in outdoor. That emphasizes the system is not affordable for outdoor use. The perception capacities of the system need to be increased to detect spatial landmarks as well.

● *Ultrasonic Cane as a Navigation Aid (UltraCane)*

Development to C-5 laser cane [37], Krishna Kumar et al. deployed an ultrasonic based cane to aid the blind people [38]. The aim of this work is to replace the laser with the ultrasonic sensors to avoid the risk of the laser. This cane is able to detect the ground and aerial obstacles.

**Figure 18:**
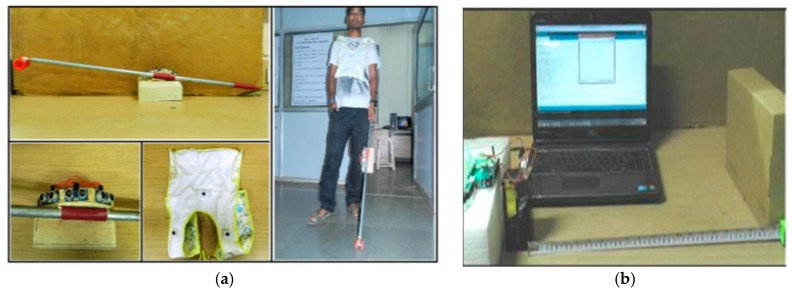
(**a**) The prototype of the device; (**b**) Detection process of the obstacle from 5 cm to 150 cm [38].

The prototype of this device as shown in Figure 18a is based on a light weight cane, three ultrasonic trans-receivers, X-bee-S1 trans-receiver module, two Arduino UNO microcontrollers, three LED panels, and pizeo buzzer. The target of the three ultrasonic sensors is to detect the ground and aerial obstacles in range of 5 cm to 150 cm. Figure 18b shows the process of the object detection within a specific distance. Once an ultrasonic wave is detected, a control signal is generated and it triggers the echo pin of the microcontroller. The microcontroller records the width of the time duration of the height of each pin and transforms it to a distance. The control signal will be wirelessly transferred by X-bee to the receiving unit which would be worn on the shoulders. The buzzer will be played to alert the user based on the obstacle’s approach (high alert, normal alert, low alert and no alert).

The authors claimed that this device can be a navigational aid to the blind people. However, the results showed it is only an object detector within a small range. Also, detection of the dynamic object was not covered in this technique which may led to an accident. In order to improve this work, tele-instructions should be giving to the user for navigation aid as well as the integration of GPS which is needed to allocate the user’s position.

● *Obstacle Avoidance Using Auto-adaptive Thresholding (Obs Avoid using Thresholding)*

**Figure 19:**
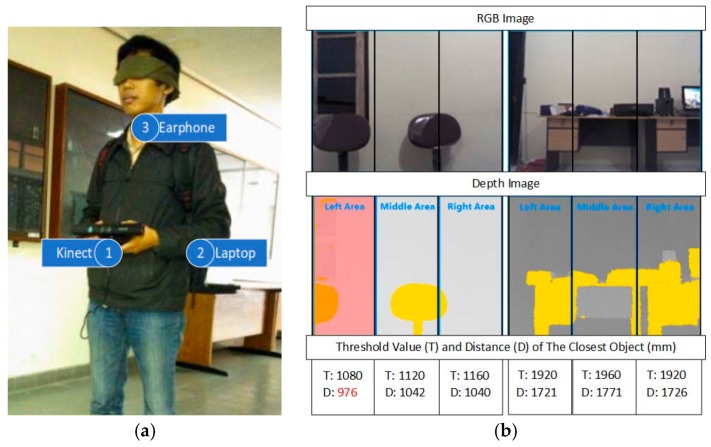
(**a**) The prototype of the proposed system; (**b**) calculating threshold value and the distance of the closest object [39].

An obstacle avoidance system using Kinect depth camera for blind people was presented by Muhamad and Widyawan [39]. The prototype of the proposed system is shown in Figure 19a. The auto-adaptive thresholding is used to detect and calculate the distance between the obstacle and the user. The notebook with USB hub, earphone, and Microsoft Kinect depth camera are the main components of the system.

The raw data (depth information about each pixel) is transferred to the system by the Kinect. To increase the efficiency, the range of a depth close to 800 mm and more than 4000 mm will be reset to zero. Then, the depth image will be divided to three areas (left, middle, and right). The auto-adaptive threshold generates the optimal threshold value for each area. Each 2 × 2 pixel area, there will be 1 pixel that is going to be used. Then, this group of data will be classified and transformed to depth histogram. Contrast function will calculate a local maximum for each depth as shown in Figure 19b. Otsu method will be applied to find the most peak threshold value [40]. Then, an average function will determine the closet object for each area afterwards. Beeps will be generated through earphone when the obstacle is in a range of 1500 mm. As it reaches 1000 mm, the voice recommendation will be produced to the blind person, so, he/she takes left, middle, or right path. The low accuracy of Kinect in closed range could reduce the performance of the system. Also, the results show the auto-adaptive threshold cannot differentiate between the objects as the distance between the user and obstacle increases.

● *Obstacle Avoidance Using Haptics and a Laser Rangefinder (Obs Avoid using Haptics&Laser)*

Using a laser as a virtual white cane to help blind people was introduced by Daniel et al. [41]. The environment is scanned by a laser rangefinder and the feedback is sent to the user via a haptic interface. The user will be able to sense the obstacle several meters away with no physical contact. The length of the virtual cane can be chosen by the user, but it is still limited. A laptop type MSI with intel core i7-740 QM, a laser rangefinder type SICK, an NVIDIA graphic card type GTX460M, and a haptic display type Novint Falcone are the main components of the proposed systems, which are structured on an electronic wheelchair. The developed software used an open source platform H3DAPI [42].

**Figure 20:**
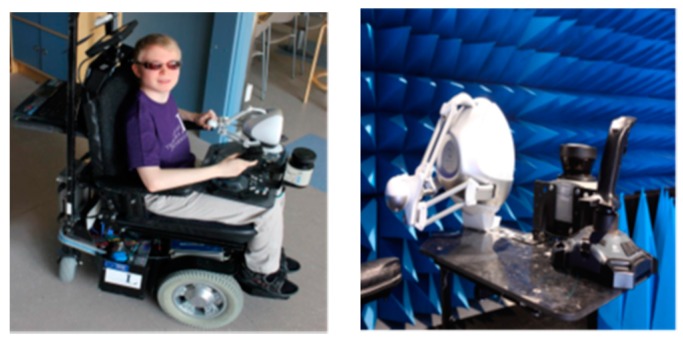
Display the proposed system mounted on the special electronic wheelchair [41].

The wheel chair will be controlled by Joystic using right hand and sensing the environment will be controlled by Falcon (haptic interface) using the other hand as shown in Figure 20. As the user starts the system, the range finder will start scanning the environment that is in front of the chair. Then, it will calculate the distance between the user and the object using the laser beams. The distance information will be transmitted to the laptop to create a 3-dimensional graph using NIVIDA card and then transmit it to the haptic device.

The results showed that the precise location of obstacles and angles were difficult to determine due to misunderstanding of the scale factor between the real and model world by the user of haptic grip translation.

● *A Computer Vision System that Ensure the Autonomous Navigation (ComVis Sys)*

A real time obstacle detection system was presented in [43] to alert the blind people and aid them in their mobility indoors and outdoors. This application works on a smartphone that is attached on the blind person’s chest. Furthermore, this paper focuses on a static and dynamic objects’ detection and classification technique which was introduced in [44].

Using detection technique in [44], the team was able to detect both static/dynamic objects in a video stream. The interested points which are the pixels that located in a cell’s center of the image are selected based on image-grid. Then, the multiscale Lucas-Kanade algorithm tracks these selected points. After that, they applied the RANSAC algorithm on these points reclusively to detect the background motion. The number of clusters are created to merge the outlines afterwards. The distance between the object and video camera defines the state of the object either as normal or urgent.

**Figure 21:**
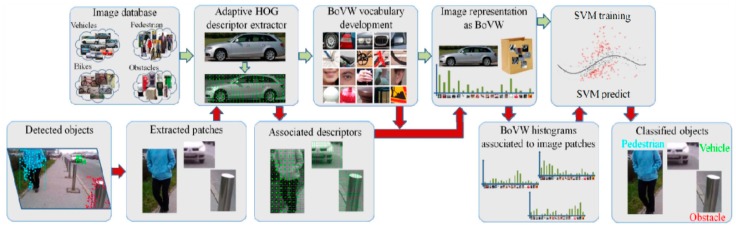
The process of detection and recognition algorithm [43].

The adapted HOG (Histogram of Oriented Gradients) descriptor was used as recognition algorithm that is integrated with the framework BoVW (Bag of Visual Words). However, the sizes of images are resizable based on the object type that the team decided. Then, they computed the descriptor on the extracted interested points of each group of images and then make clusters which contain the extracted features of all images. After that, they applied BoVW to create a codebook for all clusters (K): $W=\left\{w_{1},\;w_{2},\;\ldots ..,\;w_{k}\right\}$. Each w is a visual word that represents the system’s vocabulary. The work flow is illustrated in Figure 21. 

Now, each image is divided to blocks that created by HOG and then included into the training dataset and mapped to related visual word. At the end, they used SVM classifier for training. So, each labeled data is transmitted to the classifier to be differentiated based on specific categories.

The implementation of the system into smartphone is considered as a great mobility aid to the blind people since the smartphones nowadays are light and easy to carry. Also, using HOG descriptor to extract the feature of each set of images makes the recognition process efficient as the system not only detects the object, but also recognizes it based on its type using the clusters.

However, the fixed sizes of the image which is based on the category, can make detecting the same object with a different size a challenge. Objects in dark places and those that are highly dynamic cannot be detected. Smartphone videos are noisy as well. In addition, the tested dataset of 4500 images with dictionary of 4000 words is considered as small dataset. The system is tested and can only work on a Samsung S4.

● *Silicon Eyes (Sili Eyes)*

**Figure 22:**
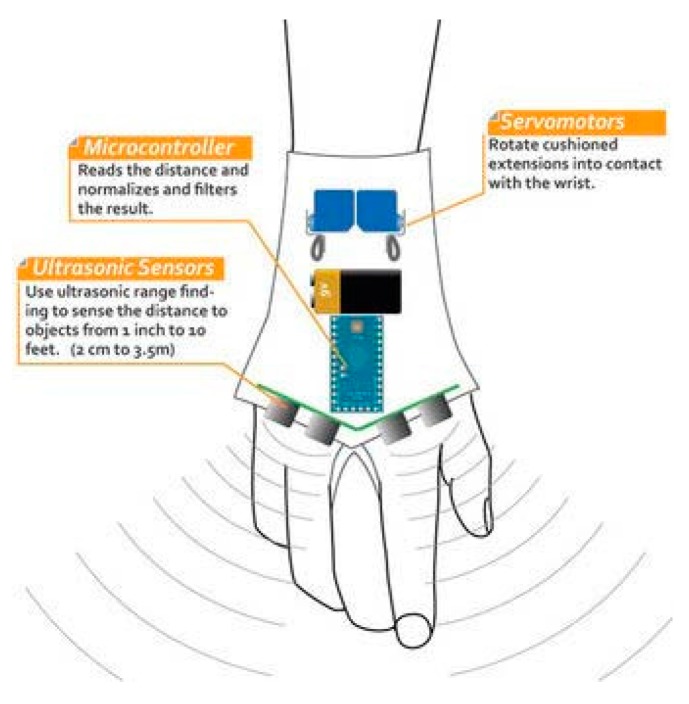
The proposed system attached on silicon glove [45].

By adapting GSM and GPS coordinator, Prudhvi et al. introduced an assistive navigator for blind people in [45]. It helps the users detect their current location, hence, navigating them using haptic feedback. In addition, the user can get information about time, date and even the color of the objects in front of him/her in audio format. The proposed device is attached within a silicon glove to be wearable as shown in Figure 22.

The prototype of the proposed device is based on a microcontroller which is 32-bit cortex-M3 to control entire system, a 24-bit color sensor to recognize the colors of the objects, light/temperature sensor, and SONAR to detect the distance between the object and the user.

The system supports a touch keyboard using Braille technique to enter any information. After the user chooses the desired destination, he/she will be directed using MEMS accelerometer and magnetometer through the road. The instructions will be sent through headset that is connected to the device via MP3 decoder. The user will be notified by SONAR on the detected distance between the user and closet obstacle. In case of emergency, the current location of the disable user will be sent via SMS to someone whose phone number is provided by the user using both technologies GSM and GPS.

The design of the system is quite comfortable as it is wearable. Also, the features that are provided to the user can give him/her more sense to the surrounded environment. However, the system needs a power tracker to keep a track of the battery. The emergency aid is not powerful as the user needs to press the button in case of the emergency and she/he has to enter phone numbers of his/her relatives, which might be a limiting factor. It would be better if the emergency feature was provided using audio messages.

● *A Path Force Feedback Belt (PF belt)*

**Figure 23:**
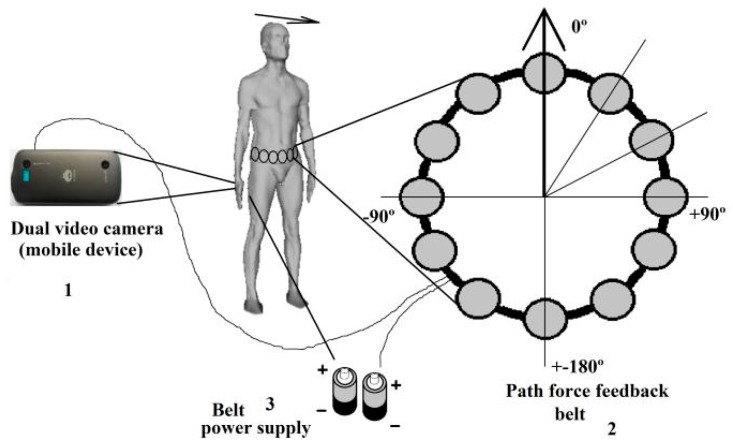
The prototype of Path Force Feedback belt design [46].

**Figure 24:**
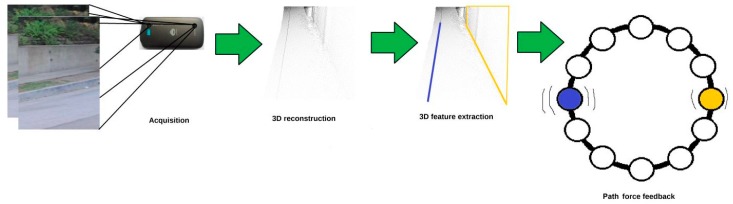
The detection process of force feedback belt [46].

A Path Force Feedback belt concept was presented by Oliveira to help blind people navigating outside through their road [46]. Figure 23 shows the three main components of the force feedback belt design; these are: the main unit (the process) with two dual video cameras, power supply which is packed into a pocket and the belt to be worn around the user’s waist. The belt has number of cells that gives a feedback to the user. The process unit uses two video cameras to take the video stream and then generates a 3D model of the user’s surrounding area as shown in Figure 24.

As the surrounding environment of the user is tracked by the processing unit in 3D model, the main features of the environment such as side walk’ borders or walls are extracted. In addition, it will aid the blind in her/his mobility by sending signals based on the extracted feature to the force feedback belt’s corresponded cells. The corresponding cells will be vibrating around the belt and show the user the right path. The system is designed such that each feature has its own signature use of the vibration pattern. So, each vibration frequency differentiates a specific feature or obstacle, e.g., the sidewalk’s border marked in blue in Figure 24. However, the user needs to be trained to distinguish the meaning of each or multiple of frequencies.

Using a 3D model within a sliding volume with continuous updating in this system provides a better and faster process of features extraction especially over the buildings and other important and urgent objects. At the same time, it can reduce the main memory consumption. Otherwise, collision awareness will perform in case of the system was disable to capture the object such as the floor.

The detection range for this design is too small as the system extracts the features of only the closest objects as explained in the paper. The blind person needs to be familiar with the surrounding area to have a proper reaction. Also, using the vibration patterns as feedback instead of audio format is not an excellent solution as the person can lose the sense of discrimination of such technique over the time; especially because there are multiple vibrations that need to be known by the user.

● *FingerReader and Eye Ring*

**Figure 25:**
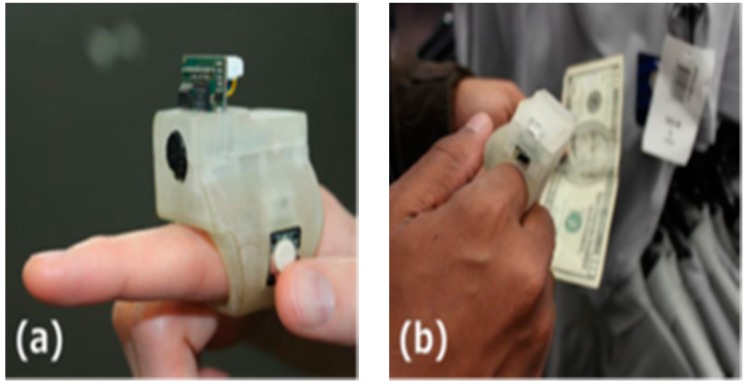
(**a**) The prototype of the EyeRing; (**b**) The process of EyeRing device of detecting and interaction application [48].

A supportive reading solution for blind people called FingerReader was introduced by Shilkrot et al. to aid disabled people in reading printed texts with a real time response [47]. This device is a wearable device on the index finger for close up scanning. So, the device scans the printed text one line at the time, then the response comes in tactile feedback and audio format. FingerReader is continuous work to EyeRing which was presented in [48] for detecting a particular object once at the time by pointing and then scanning that item using the camera on the top of the ring as shown in Figure 25.

**Figure 26:**
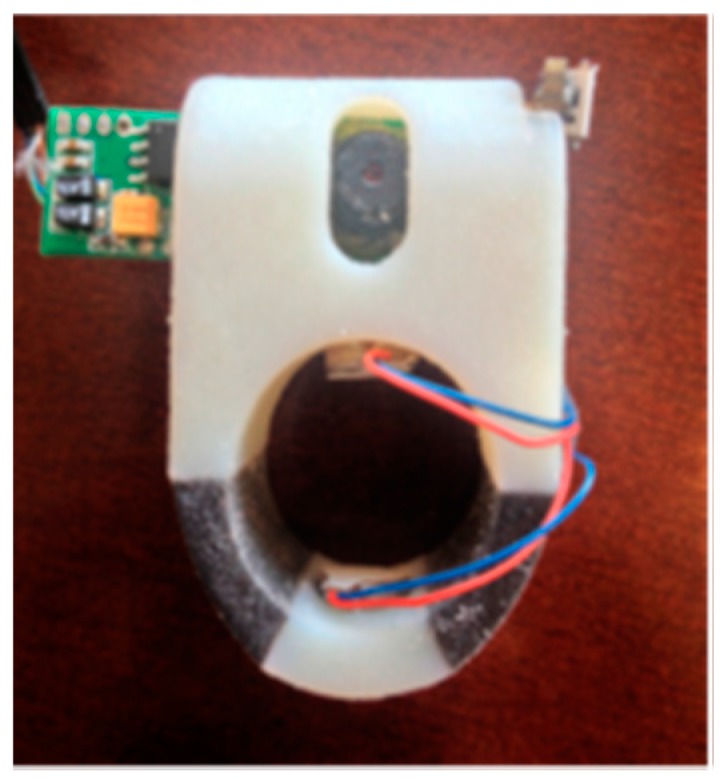
The prototype of FingerReader [47].

In this design, two vibration motors with additional multimodal feedback, dual material case for more comfort around the finger, and high resolution video stream are the expanding of the FingerReader device as shown in Figure 26. The haptic feedback was provided to guide the user to where he/she should move the camera.

**Figure 27:**
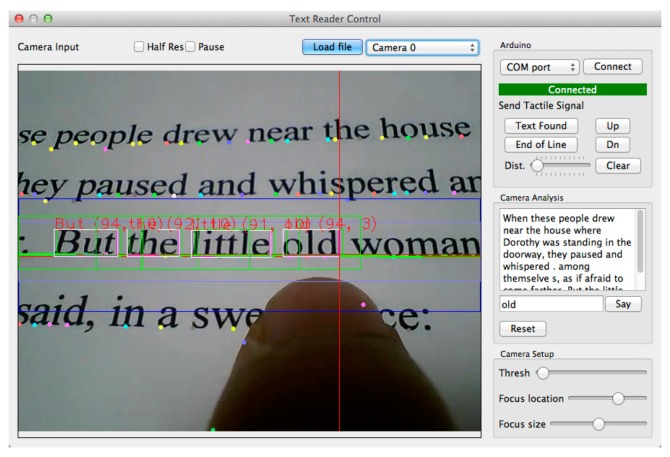
The process of the extraction and detection of printed text line [47].

The team used Text Extraction Algorithm that is integrated with Flite Text-To-Speech [49] and “ORC” [50]. The proposed algorithm extracts the printed text though close-up camera. Then, it matches the pruned curves with the lines. The duplicated words will be neglected by 2D histogram. After that, the algorithm will define the words from characters and send it to ORC. Those detected words will be saved in a template as the user continues to scan. Hence, those words will be tracked by the algorithm for any match. The user will receive an audio and haptic feedback whenever he/she sidetracks the current line. Furthermore, the user will receive signals through the haptic feedback to inform her/him about the end of the line if the system did not find any more printed text blocks. Figure 27 shows the extraction and detection process of the system.

The device was tested on four users after individual training which lasted 1 h. The feedback of the users indicated that the haptic feedback was more efficient than the audio response regarding the directions. In addition, there was a long stop between each word which confuses the user regarding what he/she should do after. However, the idea of the system is a great supportive reading solution for blind people.

● *Navigation Assistance Using RGB-D Sensor with Range Expansion (Nav RGB-D)*

**Figure 28:**
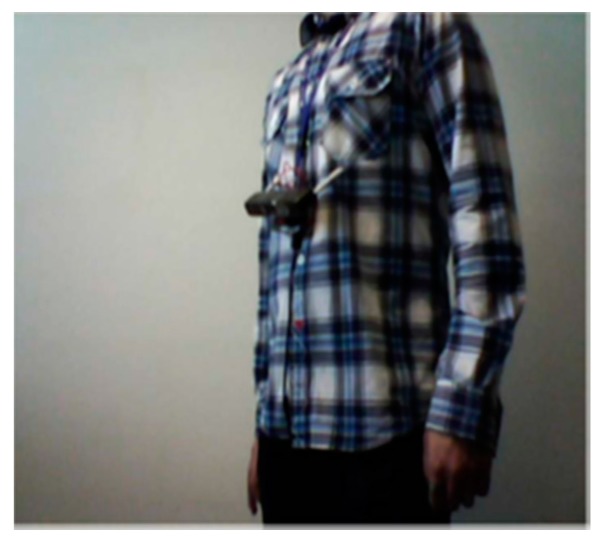
The proposed device [51].

An assistive navigator integrated both range and visual information was introduced by A. Aladren et al. to help blind people to navigate indoor areas [51]. This proposed device can be more than a navigator for blind people; it can be a light flash for anyone in dark places. This device contains two parts: one is RGB-D to obtain the color and range information between two sensors using both infrared technology and density images. The device is worn on the user’s neck as it is illustrated in Figure 28 and which connected with a laptop that is packed in a bag.

This work tries to overcome the limitation of range information by using vision computing techniques for further detection. Three steps will take place in this flow work after capturing the image by the RGB-D. The 3D point was used to extract the main features and filter all points that are represented in each cube of taken image to be one point. RANdom Sample Consensus (RANSA) is the detection algorithm, which is used to avoid any outliners as follows:

(2) $$m=\frac{\log\left(1-P\right)}{\left(1-\left(1-\varepsilon \right)p\right)}$$

**Figure 29:**
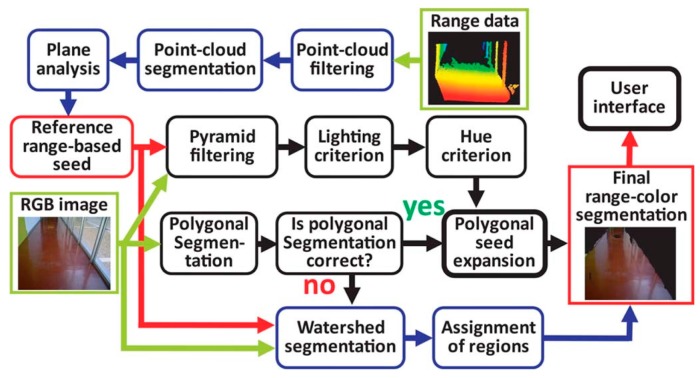
The process of the extraction and expand the range detection text [51].

In equation 2, the number of solutions in a space is *m*, and the dimension of the model is *p*, the probability of computation success is *P* and the outliners’ percentage is ε, in case of failure. These two steps will be repeated reclusively until they get the least number of points. Once the system reaches the step of classifying the object either floor or obstacle; then, the vision information technique starts to analyze the extracted cloud points based on the feature of the light, geometry and hue using the shift mean algorithm as shown in Figure 29. Based on the comparison of each extracted pixel for satisfying the similarity of above principles, they will be classified under “Floor-seed” category.

Then, they applied both the probabilistic Hough Line Transform and Canny edge detector [52] to generate board line between obstacles and floors which will be represented in polygons. Hence, based on the floor division, each region will be identified as either being floor or not. When the number of the extracted lines in the comparison is too low or too high, the watershed segmentation will be needed.

The system shows a positive performance in small places by integrating both probabilistic Hough Line Transform and Canny edge detector to classify the object as either obstacle or floor. However, the system will not provide good results when that place has a number of windows because of the infrared sensitivity to sunlight.

● *Mobile Crowd Assisted Navigation for the Visually-impaired (Mobile Crowd Ass Nav)*

A webapp over Google engine for smartphones called Mobile Crowd Assisted Navigation was developed in [53] to navigate the visually-impaired people between two points online. The aim of this framework is to offer to the user accessible, efficient and flexible crowd services for visually-impaired people. GPS, compass, accelerometer and camera are used onboard. The smartphone streams the videos and sensory information to crowd server to be used by the volunteers.

**Figure 30:**
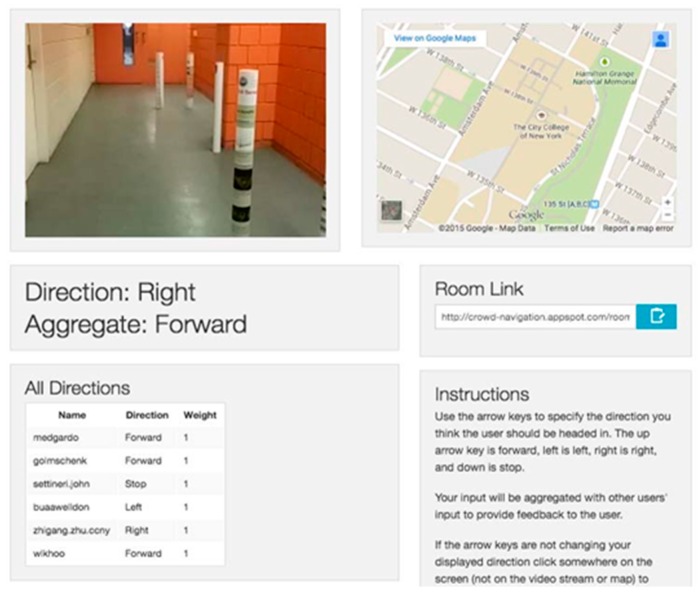
The implemented app [53].

**Figure 31:**
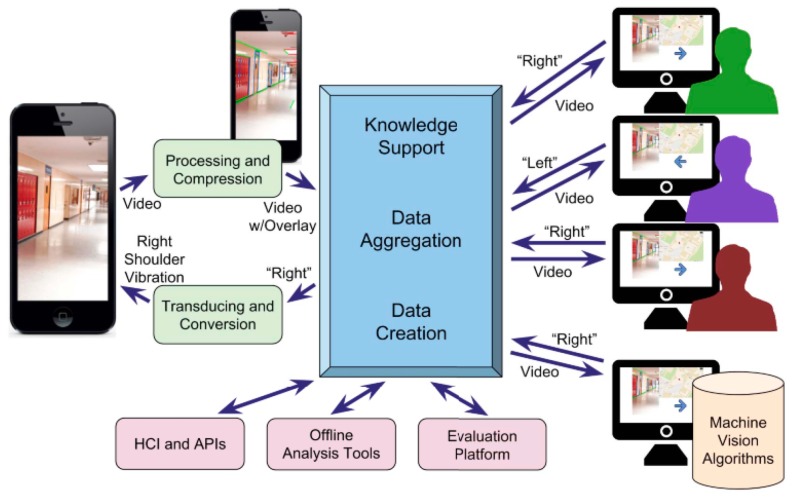
The proposed application’s dataflow [53].

The volunteers’ feedback is gathered by the Crowd program and then the system sends the final decision to the blind user through either audio format, vibration or both. The recorded video by the visual impaired user will be referred as a room and then each feedback of the volunteer will be weighted based on the accuracy of the information. The reason behind this aggregation process which is shown in Figure 30 is to eliminate the confliction of the received information about the same query from if there is more than one volunteer or if it comes from a vision algorithms machine as is shown in Figure 31.

Two experiments were tested to direct the user from one room to another using the proposed webapp and using a simple sum aggregation approach and a legion leader approach, each one at the same time. Another experiment was done on eight blind folded participates over obstacle path using the simple sum aggregation approach.

The framework can be considered as an economical solution for visually-impaired people. However, the system itself needs advanced experiments and evaluation with consideration of the delay and time alternative of aggregation process as these factors play the main roles of the system. The authors need to test the volumes of data that can be received and aggregated and how to best feed this information to the visually-impaired person.

● *A Design of Blind-guide Crutch Based on Multi-sensors (DBG Crutch Based MSensors)*

**Figure 32:**
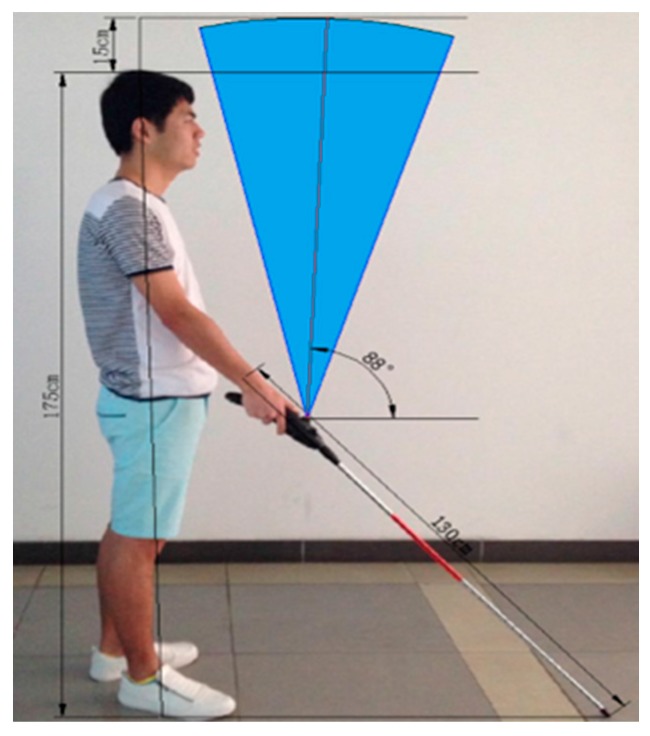
The proposed crutch with displayed detection ranges [54].

Based on the ultrasonic distance measurement approach, a guidance system for blind people was proposed in [54]. The purpose of this system is to help blind people in detecting and avoiding the obstacles in front, left front, and right front of the user as shown in Figure 32.

**Figure 33:**
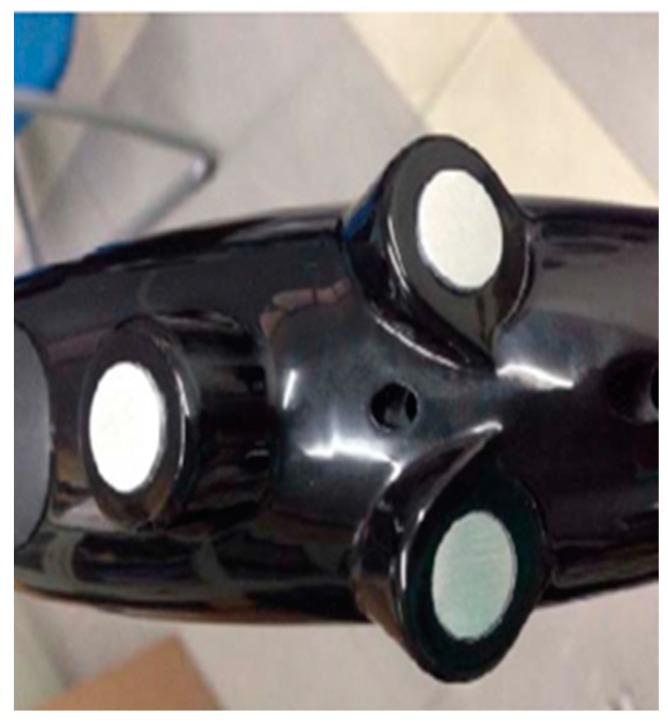
Replacement of three ultrasonic sensors on the cane [54].

Figure 33 displays the replacement of the three ultrasonic sensors on the cane. The function of these sensors is to collect the distance information from different ranges; the top sensor is used for detecting the overhead obstacle and the other two are used for detection front obstacles. In addition, ultrasonic transmitting and receiving modules, voice and vibration modules and the key to switch between the feedback modules are used in this system. The whole system is controlled by the STC15F2K60S2 microcontroller.

The STC15F2K60S2 MCU controls the signals between ultrasonic Transmit and Receive modules. The travelled times need to be recorded separately such as time1, time2 and time3 as the ultrasonic signal is emitted and the echo signals are detected. If the time counter is larger than the setup threshold, then there are no obstacles presented in that area. Based on the detected distance from the obstacle and the sensor, “the alarm decision making algorithm” produces the warning message either audio or vibration formation.

The system was successful in detecting the obstacle in four directions: front, left front, right front and overhead using three sensors. However, the detection range is small as the maximum range is 2 m. Also, the system can be considered as obstacle avoidance system, but not a navigation system as it is claimed. The feedback of this system only consists of warning messages regarding the obstacle location and there were no given directions to proceed forward.

● *Ultrasonic Assistive Headset for visually-impaired people (Ultra Ass Headset)*

**Figure 34:**
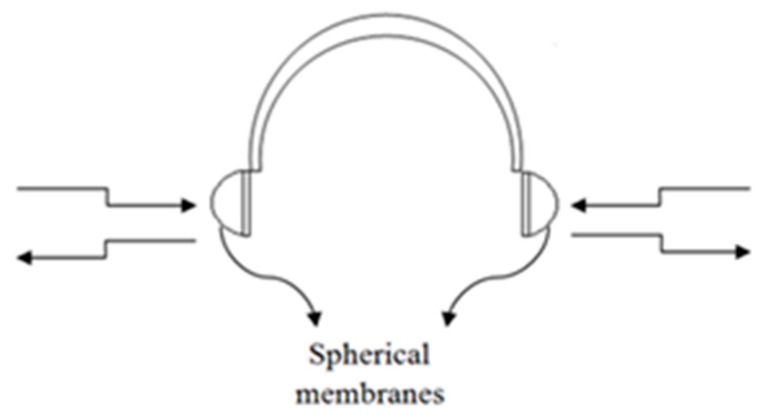
The design of ultrasonic headset [55].

An assistive headset was proposed in [55] to navigate visually-impaired people based on the ultrasonic distance measurement technology. Figure 34 illustrates the design of the ultrasonic headset which contains four ultrasonic sensors; two sensors cover each membrane to detect left and right obstacles. DYP-ME007 is the chosen type of ultrasonic sensor for a distance measurement. ISD2590 recording storage is used to record the recommended directions. There are six recorded messages, the selected information is based on the intersection of two ultrasonic sensors in case there is an obstacle.

**Figure 35:**
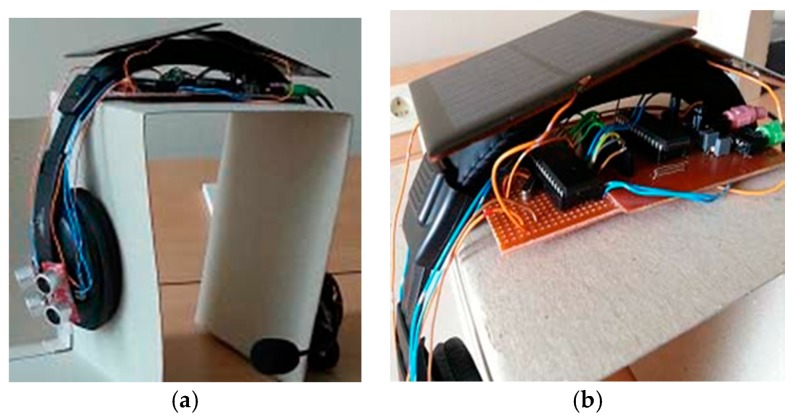
(**a**,**b**) Display the proposed ultrasonic headset with illustrating of the circuit and the solar panels [55].

The function of this system is as follows: each sensor has an ID which is produced as a binary code. Once the sensor receives a reflection of the ultrasonic wave, an output of “1” will be sent to the microcontroller, otherwise “0” will be sent. Using the binary code, the microcontroller can determine which sensor is the receiver. Based on that, the audio feedback will be played back to the user. Figure 35 shows the completed design of proposed system. 

The system is a good energy saving solution. However, the system is limited in the directions it provides to the user. Six directions cannot be sufficient enough to guide the user indoors and outdoors. Furthermore, the headset obscures the external noise, which blind people rely on to make their decision in case the system fails.

● *A Mobility Device for the Blind with Improved Vertical Resolution Using Dynamic Vision Sensors (MobiDevice Improved VerticleResolion)*

**Figure 36:**
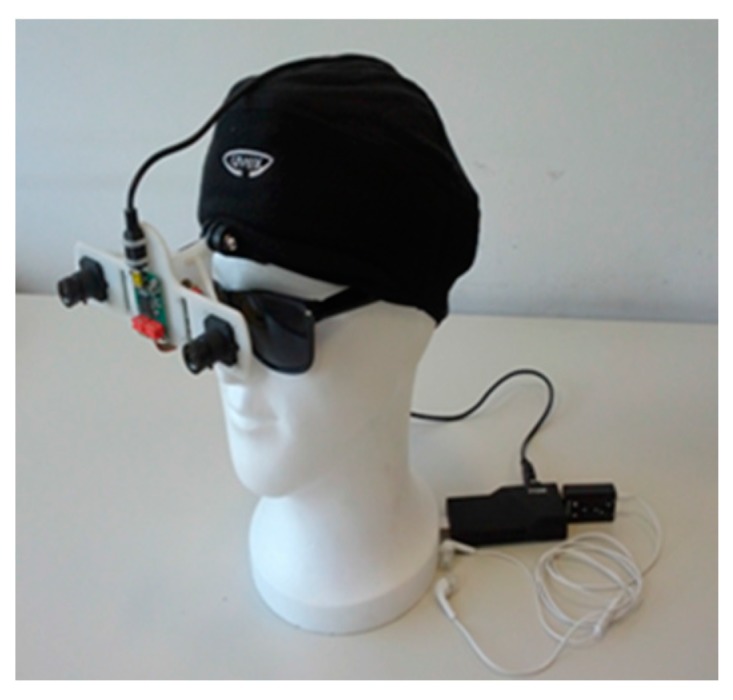
The proposed system to be mounted on the head [56].

Two retina-inspired dynamic vision sensors (DVS) were deployed in [56] to improve the mobility of visually-impaired people. Figure 36 illustrates the proposed device to be mounted on the head of the user. The aim of this work is to represent the information of the surrounding environment as an audio landscape from the simulated 3-D sound, for example MP3 format [57].

**Figure 37:**
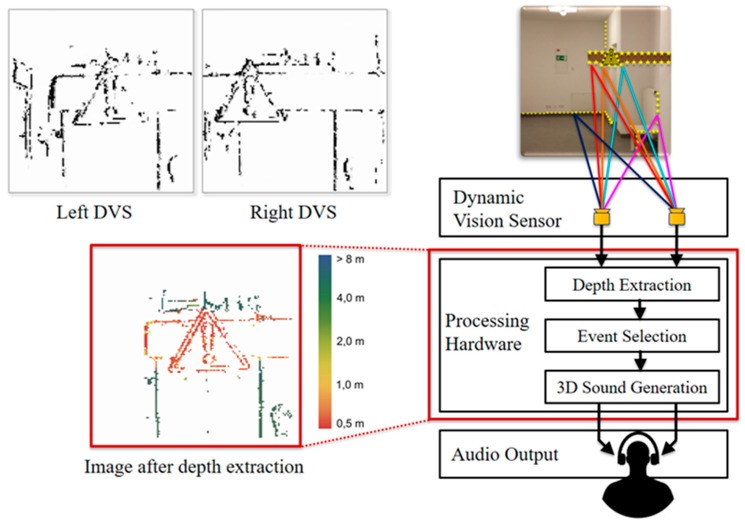
The accumulation of the interval time for forming a visual frame and the entire system is illustrated (the event distance is differentiated via colors) [56].

These sensors perform in a similar way to human retina [58,59]. So, unlike the regular cameras which are based on a fixed frame rate, DVS creates asynchronous events every time it senses an adjustment in luminance that exceeds a predefined threshold. However, the movement of the DVS can generate events at the edges of the objects or at any changed sharp textures. As a result, the accumulation of the time interval is needed in order to form a visual frame as it is illustrated in Figure 37.

As shown in Figure 37, the colors on the output of image depth extraction are represented based on the event distance. The scene is divided into three horizontal areas based on the vertical reference of that view. The middle event will be selected. Then, the event will be displayed onto simulated 3-D sound. This, in turn, will be translated to audio format to the user using the headset. The Acoustic domain was used for visual information transmission. The distance to the object can be calculated via the stereo information of DVS device.

The system was tested on two different groups to evaluate three terms which are: vertical position (up, down), object localization and horizontal position (left, right). The developed head-related transfer functions and the proposition of the focus area were used to promote resolution.

Although it is not possible to assess the object avoidance performance due to the lack of information provided by the authors, the structure of the device is comfortable and light. The system provides a power consumption solution by using less energy consumption components.

● *When Ultrasonic Sensors and Computer Vision Join Forces for Efficient Obstacle Detection and Recognition (Ultrasonic for ObstDetectRec)*

**Figure 38:**
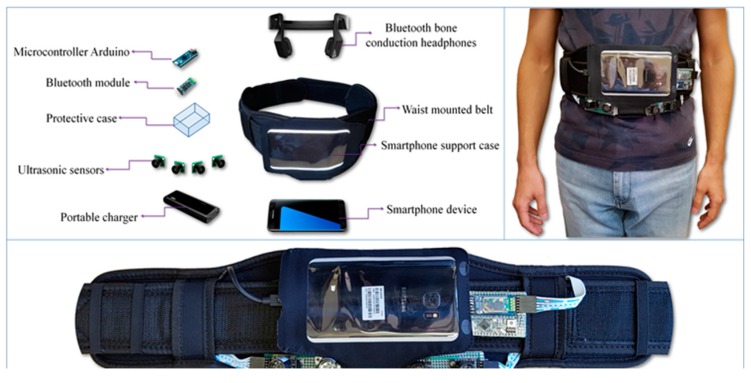
The prototype of the proposed system [60].

A wearable device was introduced in [60] to support the mobility of visually-impaired people over the civilian environment using sensors and computer vision techniques. Figure 38 illustrates the main components of the hardware architecture, whereas four ultrasonic sensors and a mobile video camera are the data sources and the smart phone is the processing unit. The device was able to identify both static and dynamic objects indoor and outdoor regardless to the object’s characters by using the machine learning and computer vision techniques. Hence, the device provides continuous information about the surrounding area through audio feedback and peeps for unrecognized objects.

**Figure 39:**
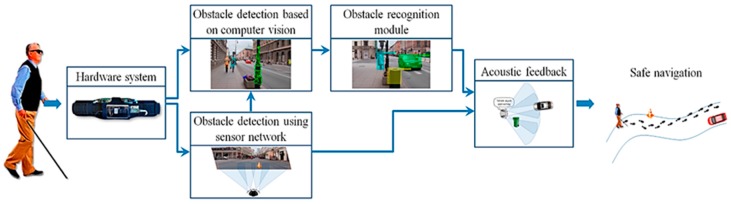
The process of the proposed navigation system [60].

Figure 39 exhibits the process of the system, where two important modules were used; obstacle detection and recognition modules. The obstacle detection module is dependent on the gathered information from both the ultrasonic sensors and smartphone camera, which will be fed to the recognition module to classify the present objects of the scene. In addition, audio feedback will be generated based on the position and distance of the object compared to the user’s position.

The integration of the proposed filter for the interested points and the points’ tracker (Lucas-Kanade) reduced the exclusion time because it requires fewer resources. Hence, RANSAC was used in order to obtain the homographic transformation between two frames of the same scene. Then, the K-mean clustering algorithm was applied to identify various dynamic objects. The detected objects were classified as urgent or normal objects. Urgent objects are those whose distance from the user is less than 2 m. Furthermore, urgent objects are the objects that are approaching the user, otherwise, they are normal objects. As a final step, the SVM classifier was integrated with CHI Square Kernel for classification training. Two thousand five hundred images were assigned for each class (four dynamic classes for outdoors) in the training stage, which is considered as a small number for accurate classification rate.

The system can be considered as a power consumption solution. Also, the integration of both the sensors network and computer vision techniques validate the robustness and reliability of the obstacle detection and recognition modules. However, the system was tested by 21 visually-impaired people. As the users are more familiar with a white cane, their feedback was that the device is not trustworthy enough and needs to be combined with the white cane. In addition, the system does not provide any navigational information and the system does not detect obstacles above the waist level.

● *SUGAR System*

The sugar system which was proposed in [61], provides visually-impaired people with guidance in an indoor environment. It provides accurate positioning information using Ultra-Wide Band technology (UWB). The system requires UWB sensors, a spatial database of the environment, a server to process the collected data, Wi-Fi connection to transmit data and a smart phone (carried by the user) to communicate with the visually-impaired person via audio feedback. UWB has a precision of up to 15 cm with a 95% confidence interval. UWB technology offers robustness because it does not need direct line of sight between tags and sensors. It uses UWB signals to acquire the person’s location and orientation. The system also has a spatial database of the environment. This spatial database is a mapping of the environment being navigated by the person.

**Figure 40:**
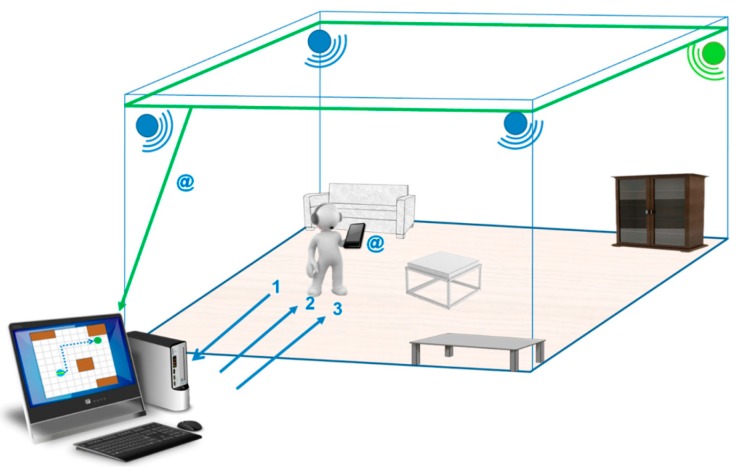
The system’s installation inside a room [61].

Other systems that use RIFD or NFC require the deployment of a number of devices to achieve the same accuracy of SUGAR. Installation of the devices in key locations is also an expensive process. The range of UWB sensors is 50 to 60 m which makes it ideal for being deployed in buildings with larger rooms. A room with a side length of 100 m requires only four UWB sensors while to achieve the same accuracy using RFID or NFC would require deployment of sensors every 80 cm. Figure 40 shows the physical components are needed for the System.

**Figure 41:**
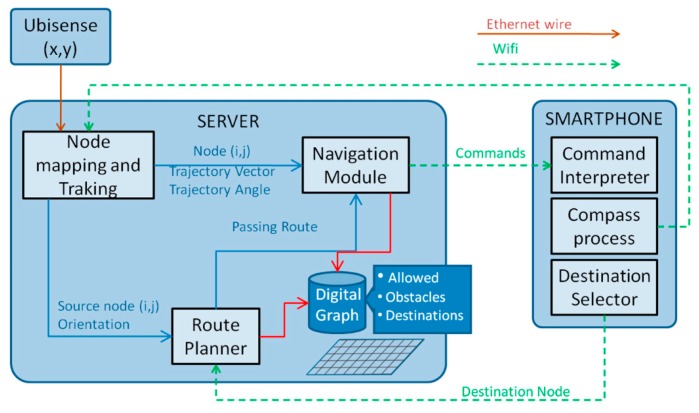
The proposed architecture [61].

We can infer the workflow of the system from the proposed architecture which is shown in Figure 41. It starts with the UWB sensors constantly tracking the person using a tag that carried by the user which will enable the system to build a Cartesian coordinate. The smartphones’ compass would also provide the person’s orientation. From the data collected, the user’s location is mapped on a graph. Once the person decides on the destination the route planner module selects the best route. As the person navigates the room, the navigation module compares their location and trajectory with the previously calculated route. The smartphone receives the commands via Wi-Fi connection and plays them back through the headphones to the person.

## 3. Analysis

In this section, we are analyzing the basic, yet the most important features for each device that we reviewed. These five features are described in Table 1. Furthermore, we are presenting here a quantitative evaluation for the reviewed systems in terms of their progress based on the main features that need to be provided by any system that offers a service for visually-impaired people.

The assistive device for a blind person needs to provide several features, among them: a clear and concise information within seconds, a consistent performance during day and night time, works indoors and outdoors; detects objects from close to further than 5 m; and detects the static and dynamic objects in order to handle any sudden appearance of objects; otherwise, the user’s life is at risk.

The evaluated features are basic and fundamental features to design an assistive device for blind people and to rely on their performance. Therefore, we give them the same weight which is 10.0, as each feature has a significant impact on the system’s performance. Based on the collected information, we gave a score for each feature of each system or device.

Since some of the evaluated systems are still in a research stage, the user’s feedback was considered in our evaluation for the devices that were tested in real scenarios only. Otherwise, our evaluation mechanism was applied based on the following criteria: the features’ are user-dependent. So, it is different from user to other. For example, some people are not interested in going outside at night, then the day/night feature is irrelevant for them. Therefore, we have weighted all the features with equal weight 10.0.

The value of each feature of each system are referred to as Vk. This value is between 0 and 10.0. The value 10.0 is assigned to a fully satisfactory feature; however, prorated values will be given to the feature in case it is not fully satisfying the criteria in Table 1. For example, we gave value 5 to a system that performs only indoors whereas it is supposed to perform indoors and outdoors, e.g., the Smart Cane. This strategy was applied for analysis type, coverage, range, time, and object type feature. However, the assigned values for range feature were applied differently, we could not give equal values for different ranges, where we are looking for devices that provide a larger detection range. So, a 2.5 value was given to those with detection range less or equal to 1 m. This range is a very low range and cannot be considered as a solution to substitute a white cane. We meant to give this low value to insist and show the importance of providing further ranges comparing to this low range.

We used the following normalization formula (equation 3) to calculate the total score for each system based on Table 2. The total score of each system in Table 2 is to give a quick evaluation on how the device is or is not satisfied. However, a full review is provided in Table A1 (Appendix A).

(3) $$Total\;Score=\overset{N}{\underset{k=0}{\sum }}\frac{10\;V_{k}}{N}+2$$

We give constant value 2 to give a clear bias in the graph and to show the clear difference between the systems and supported features. *N* refers to the total number of features of each system and *k* is the particular feature. Table 2 shows the evaluation for the most promising systems found in the literature.

## 4. Conclusions and Discussion

Table 2 shows that none of the evaluated systems was 100% satisfactorily in terms of the essential features. These features not only meet the user’s needs, but are also crucial from an engineering perspective. Those features are the main building blocks to design such a device to provide services for blind people. It is remarkable that each system supported special feature(s) over the other and might have more features than the other, but none of them supported all the evaluated features. That means we cannot consider any of them as an ideal device or system that the blind person can rely on and feel confident about using. Devices that have all the fundamental features will offer an effective performance. The reason for this limitation is that most of the researchers work on providing a new feature, but they never ensure that they support the fundamental features before they add new ones. Another reason for this is that the designers do not run enough experiments which have to be done and tested on the blind people with different scenarios to overcome any issue. The ideal device has to not only include a new feature but also to satisfy the main and basic needs of the user. The user needs to feel the sense of the surrounding environment at all times and everywhere. The system cannot be limited for specific case, otherwise, we have an incomplete design.

**Figure 42:**
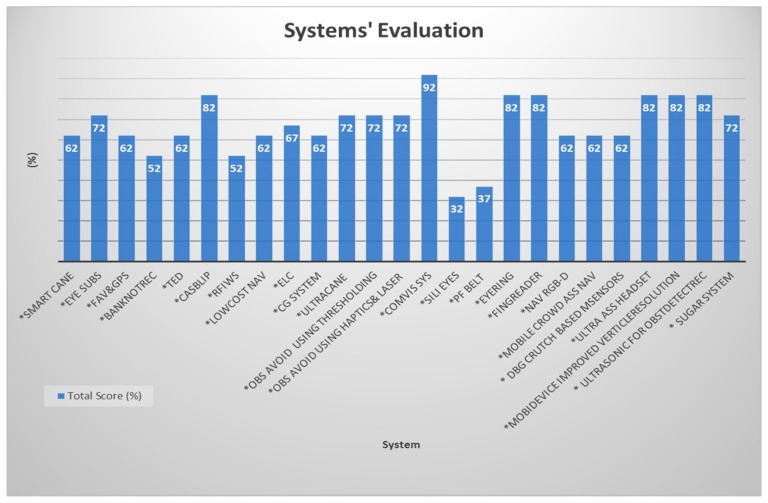
Systems’ evaluation presents the total score for each system.

Figure 42 shows us a full picture of the evaluation for each system with total score to each one. Systems with higher score demonstrate solid and improved features such as a Computer Vision System that Ensure the Autonomous Navigation (ComVis Sys) which includes most of the features. The Path Force Feedback Belt (PF belt) and other systems that have lower scores need more enhancement, yet that does not mean the value of their works is less than the systems that have higher scores. So, PF belt has score of 37% because it is not a real time (it is in the research stage): it is applied only outdoors and it is not suitable indoors, the detection range is 1 m which is considered to be a very small range and it is limited in scope. In this evaluation, we are trying to pave the road for other researchers to design devices that ensure safety and independent mobility to the visually-impaired people. The total score in Figure 42 is reflecting the giving values for each feature of each system in Table 2. In conclusion, the performance of most of the studied systems is not 100% satisfactory to the user’s need.

Our aim in this paper is to shed some light on the missing features for the most useful and significant devices. Since the technology is in advance every day, our work is to make this progress happen as early as possible. Our focus in this paper is on the performance of systems; and after careful review and study of the above systems, we developed the benchmark table (Appendix A) that includes technical perspective parameters that effected the systems’ performance and their unavailability might prevent the systems from offering the main and basic features that we discussed in Table 1. Those parameters effected the performance of the systems which should meet both the user’s needs and the engineers’ viewpoints. Both the type of the sensors used and the techniques that are used can lead to limitations if we misused them. For example, systems that used infrared technology may not have performed well during the day time due to the sensitivity of the infrared to the sunlight [62]. Whereas, systems used the Radio Frequency Identification cannot offer a large range due to the need for tags installation everywhere the system is used [63]. Also, Kinect sensor shows a small range as the accuracy of the Kinect sensor decreases as the distance between the scene and sensor increases [64,65]. In addition, the performance of ultrasonic sensors can be affected as whether the environmental parameters changed or not [66]. Hence, its maximum detection range is around 5 m. The limitation of each system is described individually in Appendix A with more comprehensive review from technical side.

Other interesting devices for blind running athletes were reviewed, but are not included in our paper due to their limited scope [67,68]. The running fields is a designed field which will not include general obstacles such as stairs. Also, the field is expected to have lines to direct the running athletes.

**Figure 43:**
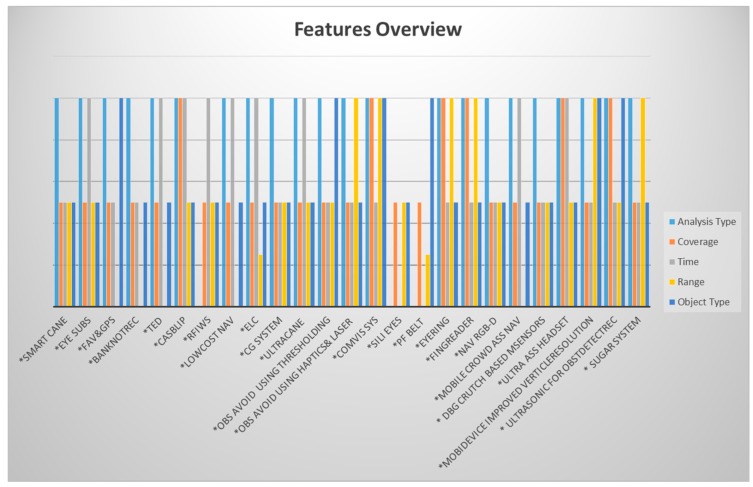
Features’ overview for each system.

As summary to our evaluation, Figure 43 shows, for every system, the penetration rate of each feature and its weight. For example, three out of the total presented systems are not real time systems, which means they are still in a research stage. Those are Sili Eyes, RFIWS, and PF belt. However, 72% of the systems have three features that are not fully satisfied. For instance, Eye Subs system provides outdoor coverage but not indoor coverage; the detection range is less than 5 m due to the ultrasonic limitation, and it detects only the static and not dynamic objects. This leads to one point that the researchers are aware of some of the fundamental features such as real time feature but not to others. So, some systems provide indoor coverage but not outdoor coverage, but the user will be in need of the system service as much indoors as outdoors, maybe even more. With this humble study, we hope that we could provide enough description of the main features that need to be included in any system that serves this group of people.

At the end of this discussion, we emphasize that this paper provides a set of essential guidelines for designing assistive devices along with the mentioned features to ensure a satisfactory performance and better computer interaction scheme with the blind person. These guidelines include:

**Performance:** all the needed functions that are listed in Table 1 should be supported.

**Wireless connectivity:** the assistive device needs to be wirelessly connected with a database to ensure information exchange.

**Reliable:** the device should meet its specification for both software and hardware.

**Simple:** simple interface and friendly operations can make the use of the device easier to the user.

**Wearable:** from our study and review, it is more flexible and comfortable to the user to wear the device rather than carry it.

**Economically accessible:** it is important to make the device economically accessible for the users in order to enhance their quality of life, otherwise, only a few people can afford it.

We are planning to continue this review by studying each function individually to overcome the mentioned weaknesses by designing an intelligent frame work that offers all the above features with more scalability and that is economically accessible.

## Figures and Tables

**Table 1:** The most important features that correspond to the user’s needs.

Feature	Description
Analysis Type	The system needs to provide a fast processing for the exchanged information between the user and sensors. For example, the system that detects the obstacle that is 2 m in front of the user in 10 s cannot be considered as real time system [12]
Coverage	The system needs to provide its services indoors and outdoors to improve the quality of visually-impaired people’s lives
Time	The system should perform as well in day time as at night time
Range	It is the distance between the user and the object to be detected by the system. Ideal minimum range is 0.5 m, whereas the maximum range should be more than 5 m. Further distance is better
Object Type	The system should avoid the sudden appearance objects, which means the system should detect the dynamic objects as the static objects

**Table 2:** Score and evaluation for each system.

System	Features					
	Real Time/not Real Time	Coverage (Indoor, Outdoor, both)	Time (Day, Night, both)	Range (R ≤ 1 m, 1 m < R ≤ 5 m, R > 5 m)	Object Type (Static, Dynamic, both)	Total Score
	Weight of 10					
*Smart Cane	10	5	5	5	5	62
*Eye Subs	10	5	10	5	5	72
*FAV&GPS	10	5	5	-	10	62
*BanknotRec	10	5	5	-	5	52
*TED	10	5	10	-	5	62
*CASBlip	10	10	10	5	5	82
*RFIWS	-	5	10	5	5	52
*LowCost Nav	10	5	10	-	5	62
*ELC	10	5	10	2.5	5	67
*CG System	10	5	5	5	5	62
*UltraCane	10	5	10	5	5	72
*Obs Avoid using Thresholding	10	5	5	5	10	72
*Obs Avoid using Haptics&Laser	10	5	5	10	5	72
*ComVis Sys	10	10	5	10	10	92
*Sili Eyes	-	5	-	5	5	32
*PF belt	-	5	-	2.5	10	37
*EyeRing	10	10	5	Specific case 10	5	82
*FingReader	10	10	5	Specific case 10	5	82
*Nav RGB-D	10	5	5	5	5	62
*Mobile Crowd Ass Nav	10	5	10	-	5	62
*DBG Crutch Based MSensors	10	5	5	5	5	62
*Ultra Ass Headset	10	10	10	5	5	82
*MobiDevice Improved VerticleResolution	10	5	5	10	10	82
*Ultrasonic for ObstDetectRec	10	10	5	5	10	82
*SUGAR System	10	5	5	10	5	72

## Appendix A

**Table A1:** Evaluation of reviewed systems based on addition features that caused that limitations of each system.

System Name/Weight/Type of Usage	Type of the Sensors	Accuracy	Analysis Type	Coverage	Measuring Angle	Cost	Limitation	Day/Night	Object Detection Range (Max/Min)	Classification Objects (Dynamic/Static)	Used Techniques for Detection, Recognition or Localization
*Smart Cane* *Weight: N/A* *Type of usage: pilot stage*	Ultrasonic sensors Water detector	N/A	Real time	Outdoor (only areas have RFID tags)	N/A	High	The water sensor can’t detect the water if it is less than 0.5 deep. The buzzer won’t stop before it is dry. A power supply meter reading needs to be installed to track the status	Day	1 m–1.5 m	Static	Ultrasonic technology
*Eye Substitution* *Weight: light* *Type of usage: pilot stage*	2 Ultrasonic Sensors Vibrator motors	N/A	Real time	Outdoor	Each sensor has a cone angle of 15°	$1790	The design of the system is uncomfortable due to the wood foundation which will be carried by the user most of the time as well as and the figures holes. The team used 3 motors for haptic feedback. They could use a 2-d array of such actuators that can give feedback about more details. Limited use by only Android devices	Day/Night	2 m–3 m	Static	GPS, GSM, and GPRS Ultrasonic technology
*Fusion of Artificial Vision and GPS* *Weight: N/A* *Type of usage: deployment stage*	Optical Sensors Bumble bee Stereo Camera 3-axis Accelerometers Electronic compass Pedometer	Accurate results for user position	Real time	Outdoor	6° of visual angle with (320 × 240 pixel) and 100° field of view with (640 × 480 pixel)	low	The system was tested on the function of the object’s avoidance technique. The system has not been tested or integrated with navigation systems to insure its performance; whether it will enhance the navigation systems as the authors promised or not is unknown.	Day	2 m–10 m	Static/dynamic	Global Position System (GPS), Modified Geographical Information System (GIS) and vision based positioning SpikNet was used as recognition algorithm
*Banknote Recognition* *Weight: N/A* *Type of usage: pilot stage*	iV-CAM	80%	Real time	N/A	N/A	low	This device was tested only on the Thai banknotes and coins, and it is not capable of working on other currencies that have similar colors of banknotes or similar sizes of coins. The device needs a method that controls the natural light that is used	Day	Closed View	Static	RGB model Banknotes Classification Algorithm
*TED* *Weight: light* *Type of usage: pilot stage*	Detective Camera	The corresponds are based on the feeling on the dorsal part of the tongue, (1,2,3,4) 100% (7) 10% (5,6,8) 50%	Real time	Outdoor	N/A	low	Antenna is not omni-directional. The range of voltage is not enough to supply the device. It is more difficult to recognize the pulses on the edges of the tongue.	Day/Night	N/A	Static	Tongue–Placed Electro tactile Display
*CASBlip* *Weight: N/A* *Type of usage: pilot stage*	3D CMOS sensor	80% in range of 0.5 m–5 m and less than 80% with further distance	Real time	Indoor/outdoor	64° in azimuth	N/A	Small detection range Image acquisition technique needs more than 1X64 CMOS image sensor. Acoustic module needs to be improved (it can add sounds in elevation)	Day/Night	0.5 m–5 m	Static	Binaural Acoustic module Multiple double short-time integration algorithms (MDSI)
*RFIWS* *Weight: N/A* *Type of usage: research stage*	None	N/A	Not-Real time	Outdoor	N/A	N/A	Collision of RFID Each tag needs specific rang which needs to be tested separated (scoop limitation) The tags cannot read the radio waves if case these tags get wrapped up or covered.	Day/Night	1 m–3 m	Static	Ultra-high frequency (UHF)
*A Low Cost Outdoor Assistive Navigation System* *Weight: N/A* *Type of usage: pilot stage*	3 Axial accelerometer sensors Magnetometer sensor	Good accuracy within residential area, but not as in an urban environment	Real time	Outdoor	N/A	$138	The accuracy of GPS receiver in high rise building is degraded. Limited scope, the GPS receiver needs to be connected via Bluetooth to perform.	Day	N/A	Static	GPS technology Geo-Coder-US Module MoNav ModuleBluetooth
*ELC* *Weight: 0.170 Kg* *Type of usage: deployment stage*	Ultrasonic sensor Micro-motor actuator	N/A	Real time	Outdoor	N/A	N/A	It is a detector device for physical obstacles above the waist line but the navigation still relies on the blind person.	Day/Night	Close objects over the waistline	Static	Ultrasonic sensor technology Haptics and tactile techniques
*Cognitive Guidance System* *Weight: N/A* *Type of usage: pilot stage*	Kinect sensor Video camera stereo Imaging sensor sonny ICx424 (640 × 480) RBG-D sensor for 3D point	N/A	Real time	Indoor	180°	N/A	Only 49 Fuzzy rules were covered which cover 80 different configurations. The perception capacities of the system need to be increased to detect spatial landmarks. Improve the stabilization of reconstructed walking plane and its registration through the frame.	Day	1.5 m–4.0 m	Static	The Canny filter for edge detection. Stereo vision, vanishing point and fuzzy rules (fuzzy logic and Mandani fuzzy decision system) to infer about the distances of objects.
*Ultrasonic Cane as a Navigation Aid* *Weight: light* *Type of usage: pilot stage*	Ultrasonic sensor (trans-receiver) Arduino UNO microcontroller wireless X-bee S1 trans receiver module	N/A	Real time	Indoor	30°	N/A	Just an object detector Small detection rang Does not detect objects that suddenly appear	Day/Night	5–150 cm	Static	Ultrasonic Technology
*Obstacle Avoidance Using Auto-adaptive Thresholding* *Weight: N/A* *Type of usage: pilot stage*	Kinect’s depth camera	N/A	Real time	Indoor	Horizontal 57.50° and Vertical 43.5°	N/A	The accuracy of Kinect depth image decreases when the distance between the scene and sensor increase. Auto-adaptive threshold could not differentiate between the floor and the object after 2500 mm. That increases the average error of distance detection. The depth camera has to be carried which is a lot of load on the user’s hand.	Day	0.8 m–4 m	Static/dynamic	Auto-adaptive Thresholding (divides equally a depth image into three areas. It finds the most optimal threshold value automatically (auto) and vary among each of those areas (adaptive).
*Obstacle Avoidance Using Haptics and a Laser Rangefinder* *Weight: N/A* *Type of usage: pilot stage*	Basely the system was built on the use of laser but the Novint Falcon has Encoder LED emitters and photo sensors Supplementary Sensors	N/A	Real time	Indoor	Horizontal 270° in front of chair	N/A	Precise location of obstacles and angles were difficult to determine.	Day	20 m with 3 cm error	Static	Haptics and a Laser Rangefinder
*A Computer Vision System that Ensure the Autonomous Navigation* *Weight: N/A* *Type of usage: deployment stage*	Monocular camera	High Accuracy	Real time	Indoor/outdoor	Angular field of camera view of 69°	*low*	Their fixed sizes of the image based on the category can make detecting the same object with different sizes a challenge. Since the proposed system is based on a smartphone video camera; if the video camera is covered by the blind person’s clothes, then the system cannot work. The objects are in dark places and highly dynamic objects cannot be detected. The overhead and noise of smartphones videos. The tested dataset of 4500 images and dictionary of 4000 words are considered as a small dataset. The system is tested and it works only on a Samsung S4 which makes it limited in scope.	Day	Up to 10 m	Static/Dynamic	Lucas–Kanade algorithm and RANSAC algorithm are used for detection. Adapted HOG descriptor extractor, BoVW vocabulary development and SVM training are used for recognition.
*Silicon Eyes* *Weight: N/A* *Type of usage: research stage*	24-bit color sensor SONAR obstacle detection light sensor 3-axis MEMS magnetometer 3-axis MEMS Accelerometer	N/A	Not-Real time	Not tested	N/A	N/A	A power supply meter reading needs to be installed to track the status. Low accuracy of GPS receiver in high rise buildings. The haptic feedback is not efficient. Limited memory of 2 GB micro-SD card to save user information.	Not tested	2.5 cm–3.5 m	Static	GPS & GSM technology
*A Path Force Feedback Belt* *Weight: N/A* *Type of usage: research stage*	IR sensor Two depth sensors (sensor 2 dual video cameras type Kinect)	N/A	Not-Real Time	Outdoor	360° over the blind’s waist	N/A	The detection range for this design is too small. The user needs to be trained in differentiating the vibration patterns for each cell. Using vibration patterns as feedback instead of audio format is not an excellent solution as the person can lose the sense of discrimination of such techniques over the time.	Not tested	Short	Static/dynamic	Infrared technology and GPS
*EyeRing* *Weight: N/A* *Type of usage: pilot stage*	Atmel 8 bit microcontroller OV7725 VGA CMOS sensor for image acquisition	N/A	Real time	Indoor/outdoor	Not Applicable	N/A	The system does not provide a real time video feedback. The system is limited to single object detection, which cannot be very useful to the disabled person.	Day	Close up view	Static	Roving Networks RN-42 Bluetooth module
*FingerReader* *Weight: N/A* *Type of usage: pilot stage*	Atmel 8 bit microcontroller OV7725 VGA CMOS sensor for image acquisition Vibration motors	93.9%	Real time tactile feedback20 m processing time	Indoor/outdoor	Not Applicable	N/A	There is a real time response for the audio feedback, but there is a long stop between the instructions. Also, the system prototype contains two pieces one is the ring, the other is the computation element which need to be carried all the time by the user for I/0 speech, otherwise the user will not be able to receive the feedback.	Day	Close up view	Static	Roving Networks RN-42 Bluetooth module
*Navigation Assistance Using RGB-D Sensor With Range Expansion* *Weight: N/A* *Type of usage: pilot stage*	RGB-D sensor	95%	Real time	Indoor	*N/A*	low	The effective of the infrared to the sunlight can negatively affect the performance of the system outdoors and during the day time.	Night	Up to 3 m using range information technique and from 3 m and further using the vision information	Static	RANdom Sample Consensus (RANSA) detection algorithm Image intensities and depth information (computer vision) Infrared technology and density images
*Mobile Crowd Assisted Navigation for the Visually-impaired* *Weight: N/A* *Type of usage: pilot stage*	Camera GPS Compass Accelerometer	20.5% improvement in crowd sound for navigation	Real time	Indoor	N/A	N/A	The collected information is based on the volunteers’ availability. There is a possibility of no input in the interval time which fails the goal of the service.	Day/Night	N/A	Dynamic	Crowd sounding service through Goagle engine for navigation Machine vision algorithm
*A Design of Blind-guide Crutch Based on Multi-sensors* *Weight: N/A* *Type of usage: deployment stage*	3 Ultrasonic sensors	N/A	Real time	Outdoor	30° detection range for 2 sensors, 80° detection range for overhead	N/A	The detection range is small. This system is claimed to be navigation system, however, there are no given directions to the user.	Day	0 m–2 m in front	Static	Ultrasonic distance measurement approach
*Ultrasonic Assistive Headset for visually-impaired people* *Weight: light* *Type of usage: pilot stage*	4 Ultrasonic type (DYP-ME007) sensor obstacle detector	N/A	Real time	Indoor/outdoor	60° between ultrasonic distance sensors	N/A	Limited directions are provided. The headset obscures the external noise.	Day/Night	3 cm–4 m	Static	Ultrasonic technology
*A Mobility Device for the Blind with Improved Vertical Resolution Using Dynamic Vision Sensors* *Weight: light* *Type of usage: pilot stage*	2 retine-inspired dynamic vision sensors (DVS)	99% ± object detection, 90% ± 8% horizontal localization, 96% ± 5.3% size discrimination	Real time	Indoor	N/A	low	The modules are very expensive. Further intensive tests need to be done to show the performance object avoidance and navigation techniques, whereas, the test was mainly on object detection technique for the central area of the scene.	Day	0.5 m–8 m	Dynamic/static	Event-based algorithm
*Ultrasonic for ObstDetectRec* *Weight: 750 gram* *Type of usage: pilot stage*	4 ultrasonic sensors (Maxsonar LV EZ-0)	N/A	Real time	Indoor/outdoor	±40°	Low	The system cannot detect obstacles above waist level. There is no navigational information provided. Small detection range. It is not an independent device.	Day	2 < R ≤ 5 m	Static/dynamic	Vision-based object detection module. Ultrasonic technology. SVM
*SUGAR system* *Weight: N/A* *Type of usage: pilot stage*	Ultra-wide band Sensors(UWB)	High Accuracy	Real time	Indoor	N/A	N/A	Sensors would have to be deployed in every room. The room has to be mapped beforehand. User needs to select destination beforehand. It is not suitable for outside use.	Day (the system was not tested for night time)	50 m–60 m	static	UWB positioning technique Path Finding Algorithm Time Difference of Arrival technique (TDOA)

* Not Available online: N/A.
